# Distinct features of the *Leishmania* cap-binding protein LeishIF4E2 revealed by CRISPR-Cas9 mediated hemizygous deletion

**DOI:** 10.1371/journal.pntd.0008352

**Published:** 2021-03-24

**Authors:** Nofar Baron, Nitin Tupperwar, Irit Dahan, Uzi Hadad, Geula Davidov, Raz Zarivach, Michal Shapira

**Affiliations:** 1 Department of Life Sciences, Ben-Gurion University of the Negev, Beer Sheva, Israel; 2 Centre for Cellular and Molecular Biology, Hyderabad, India; 3 Ilse Katz Institute for Nanoscale Science and Technology, Ben-Gurion University of the Negev, Beer Sheva, Israel; Instituto Oswaldo Cruz, BRAZIL

## Abstract

*Leishmania* parasites cycle between sand-fly vectors and mammalian hosts adapting to alternating environments by stage-differentiation accompanied by changes in the proteome profiles. Translation regulation plays a central role in driving the differential program of gene expression since control of gene regulation in *Leishmania* is mostly post-transcriptional. The *Leishmania* genome encodes six eIF4E paralogs, some of which bind a dedicated eIF4G candidate, and each eIF4E is assumed to have specific functions with perhaps some overlaps. However, LeishIF4E2 does not bind any known eIF4G ortholog and was previously shown to comigrate with the polysomal fractions of sucrose gradients in contrast to the other initiation factors that usually comigrate with pre-initiation and initiation complexes. Here we deleted one of the two LeishIF4E2 gene copies using the CRISPR-Cas9 methodology. The deletion caused severe alterations in the morphology of the mutant cells that became round, small, and equipped with a very short flagellum that did not protrude from its pocket. Reduced expression of LeishIF4E2 had no global effect on translation and growth, unlike other LeishIF4Es; however, there was a change in the proteome profile of the LeishIF4E2(+/-) cells. Upregulated proteins were related mainly to general metabolic processes including enzymes involved in fatty acid metabolism, DNA repair and replication, signaling, and cellular motor activity. The downregulated proteins included flagellar rod and cytoskeletal proteins, as well as surface antigens involved in virulence. Moreover, the LeishIF4E2(+/-) cells were impaired in their ability to infect cultured macrophages. Overall, LeishIF4E2 does not behave like a general translation factor and its function remains elusive. Our results also suggest that the individual LeishIF4Es perform unique functions.

## Author summary

*Leishmania* parasites cause a broad spectrum of diseases with different pathological symptoms. During their life cycle the parasites shuffle between sand-fly vectors and mammalian hosts adapting to the changing environments via a stage specific program of gene expression that promotes their survival. Translation initiation plays a key role in control of gene expression and in *Leishmania* this is exemplified by the presence of multiple cap-binding complexes that interact with mRNAs. The parasites encode multiple paralogs of the cap-binding translation initiation factor eIF4E and of its corresponding binding partner eIF4G forming complexes with different potential functions. The role of LeishIF4E2 remains elusive: it does not bind any of the LeishIF4G candidate subunits and associates with polysomes, a feature less common for canonical translation factors. Here we generated a hemizygous *Leishmania* mutant of the least studied cap-binding paralog, LeishIF4E2, by eliminating one of the two alleles using the CRISPR-Cas9 methodology. The mutant showed morphological defects with short and rounded cells, and a significant reduction in their flagellar length. Moreover, the LeishIF4E2(+/-) cells were impaired in their ability to infect cultured macrophages. The mutants showed differences in their proteome: upregulated proteins were related mainly to general metabolic processes including enzymes involved in fatty acid metabolism, DNA repair and replication, signaling, and cellular motor activity. Downregulated proteins included flagellar rod and cytoskeletal proteins, as well as surface antigens involved in virulence. Overall, LeishIF4E2 does not behave like a general translation factor and its function remains elusive. It could affect translation of a particular set of transcripts, causing direct or downstream effects that do not affect global translation. Our results suggest that individual LeishIF4Es perform specific functions.

## Introduction

*Leishmania* species are unicellular trypanosomatid protists that display a digenetic life cycle, migrating between sand flies and mammals [1]. The parasites reside in the alimentary canal of the sand fly vector as extra-cellular promastigotes attached to the gut wall. Promastigotes are elongated cells equipped with a flagellum that enables them to attach to the gut wall and to subsequently migrate towards the front parts of the mouth during metacyclogenesis [1]. Metacyclic promastigotes are transmitted into the mammalian host during the females’ blood meal, where they enter macrophages and differentiate into non-flagellated amastigotes that reside within phagolysosomal vacuoles. Amastigotes are smaller in size, round and non-motile as their flagellum does not protrude from the flagellar pocket. During their life cycle *Leishmania* parasites are exposed to non-favorable environments and extreme conditions which are known to induce a developmental program of gene expression [2,3].

Translation in eukaryotes proceeds mostly by cap-dependent mechanisms which are primarily controlled at the level of initiation [4]. As part of this process, an eIF4F complex that consists of the cap-binding protein (eIF4E), an RNA-helicase (eIF4A) and a scaffold protein (eIF4G) that holds together the pre-initiation complex (PIC), binds to the mRNA. Assembly of the cap-binding complex is globally controlled by 4E-binding proteins, such as 4E-BP, a small (10 KDa) unstructured protein that contains the consensus Y(X_4_)LΦ motif for binding to eIF4E. 4E-BP competes with eIF4G for binding to eIF4E thereby preventing assembly of the translation initiation complex [5]. Other 4E-binding proteins can control the cap-dependent translation either globally, or in a transcript-specific manner, when an inhibitory complex that assembles over the cap-binding protein is stabilized through proteins that interact with elements in the 3’ UTR [6,7]. Another mode of regulation involves an ortholog of eIF4E, denoted 4E-HP, which binds to the cap-structure, but not to any eIF4G, competing with the canonical eIF4E to bind the mRNA cap-structure [8].

There are six paralogs of the cap-binding protein eIF4E in *Leishmania*, designated LeishIF4E 1–6. They vary in their cap-binding affinities and were reported to have diverse functions [9,10]. Three paralogs of eIF4A were reported in *Leishmania*, [11] and five eIF4G candidates containing the MIF4G domain (the middle domain of eIF4G) have been identified [9,11,12]. Among the six LeishIF4Es, only four of the paralogs bind specific LeishIF4Gs, whereas LeishIF4E1 and LeishIF4E2 have no LeishIF4G binding partners. However, unlike LeishIF4E1 which is expressed and functional in all life forms, expression of LeishIF4E2 is dramatically reduced in axenic amastigotes [13].

LeishIF4E4 was suggested to be a canonical translation factor in promastigotes, forming a cap-binding complex with LeishIF4G3 and LeishIF4A [14]. LeishIF4E1 is the only cap binding protein that maintains its high expression and efficient binding to m^7^GTP in axenic amastigotes [13]. A viable null mutant of LeishIF4E1 generated by CRISPR-Cas9 displayed a reduction in global translation, suggesting that despite the absence of a functional eIF4G binding partner, LeishIF4E1 functions in promoting translation [15]. LeishIF4E3 binds LeishIF4G4, one of the five MIF4G proteins in *Leishmania* and *Trypanosoma* [10,16,17]. However, upon nutritional stress LeishIF4E3 concentrates in cytoplasmic granules that function as storage sites for inactive mRNAs and ribosomal proteins [18]. Deletion of a single LeishIF4E3 copy by CRISPR-Cas9 led to changes in cell morphology and infectivity; it was not possible to generate a null mutant suggesting that LeishIF4E3 is an essential gene [19]. Two additional cap-binding proteins were identified in *T*. *brucei*. TbIF4E5 interacts with TbIF4G1 and TbIF4G2 and has a role in parasite motility although the role of each individual complex is not yet clear [20]. TbIF4E6 forms a tripartite cytosolic complex with TbIF4G5 and with a hypothetical protein, TbG5-IP, that has domains typical of capping enzymes. Although the exact role of this complex unknown, it may be involved in recovery of decapped mRNAs [21].

LeishIF4E2 is the least studied paralog in *Leishmania*. It contains the three conserved tryptophan residues that are part of the cap-binding pocket and binding assays based on recombinant LeishIF4E2 showed that it binds better to cap-4 than to m^7^GTP [10]. LeishIF4E2 co-migrated with high MW fractions on sucrose gradients. Treatment of the cell extracts with mung-bean nuclease shifted the migration of LeishIF4E2 to the top of the gradient suggesting that it was associated with polysomes [10]. Previous analyses of the canonical translation initiation factors in eukaryotes showed that the preinitiation complexes concentrated mostly in the 43S fractions on sucrose gradients [22]; however, LeishIF4E2 was rather different as it migrated with the heavy fractions.

The *T*. *brucei* ortholog of LeishIF4E2, TbEIF4E2, also fails to bind to any TbEIF4G homolog [23]. However, it binds mature mRNAs and associates with one of the two histone mRNA stem-loop binding proteins expressed in *T*. *brucei* (SLBP2). Both SLBP2 and TbEIF4E2 are cytoplasmic proteins which are more abundant in early-log phase cells [23]. However, the role of TbEIF4E2 in translation remains vague. Here we describe a hemizygous mutant of the *Leishmania* ortholog, LeishIF4E2, that was generated by CRISPR-Cas9. Deletion of a single LeishIF4E2 allele reduced its expression, leading to phenotypic effects that may shed a light on its functions.

## Materials and methods

### Cells

*Leishmania mexicana* M379 cells were routinely cultured at 25 ^o^C in Medium 199 (M199), pH 7.4, supplemented with 10% fetal calf serum (FCS, Biological Industries), 5 μg/mL hemin, 0.1 mM adenine, 40 mM Hepes, 4 mM L-glutamine, 100 U/mL penicillin and 100 μg/mL streptomycin.

RAW 264.7 macrophage cells were grown at 37 ^o^C in DMEM supplemented with 10% FCS, 4 mM L-glutamine, 0.1 mM adenine, 40 mM Hepes pH 7.4, 100 U/mL penicillin and 100 μg/mL streptomycin, in an atmosphere of 5% CO2.

### Affinity purification of recombinant LeishIF4E2

The open reading frame (ORF) of LeishIF4E2 was amplified with primers LeishIF4E2-His forward and LeishIF4E2-His reverse (S1 Table) and cloned in pET28 vector and expressed in the Rossetta strain of *E*. *coli*. The bacterial cells were grown to OD_600_ of 0.5–0.7 and expression was induced by the addition of 0.3 mM IPTG at 25 ^o^C for 8 hrs. The cells were harvested and re-suspended in lysis buffer [20 mM HEPES-KOH pH 7.5, 100 mM KCl, 2 mM Tris (2-carboxyethyl) phosphine hydrochloride (TCEP), 0.1 mM EDTA, 0.01% Triton X-100, a cocktail of protease inhibitors (Sigma) and 5 μg/mL DNaseI. Ni-NTA beads (Cube Biotech, 5 mL) were pre-washed with two column volumes (CVs) of Binding Buffer [20 mM HEPES-KOH pH 7.5, 150 mM KCl, 2 mM TCEP]. The cells were disrupted using a French Press at 1500 psi, followed by centrifugation at 45,000 rpm (Beckman 70 Ti rotor) for 45 min. The supernatant was further incubated with Ni-NTA beads that were washed four times with two CVs of Wash Buffers (WB) containing 20 mM Hepes-KOH pH 7.5, 2 mM TCEP, 10 mM imidazole, in 2 CVs and gradually decreasing KCL concentrations (750 mM, 500 mM, 250 mM and 100 mM). Finally, the beads were eluted with a single CV of PBS containing 300 mM imidazole in 20 mM Hepes-KOH pH 7.5, 100 mM KCl, 2 mM TCEP. The recombinant protein was dialyzed overnight at 4 ^o^C against the Binding Buffer.

### CRISPR-Cas9 mediated deletion of a single allele of LeishIF4E2

Plasmids developed for the CRISPR system in *Leishmania* were obtained from Dr. Eva Gluenz (University of Oxford, UK, [24]). The pTB007 plasmid used contained the genes encoding the *Streptococcus pyogenes* CRISPR-associated protein 9 endonuclease and the T7 RNA-polymerase (Cas9/T7), along with the hygromycin resistance gene. pTB007 was transfected into *L*. *mexicana* promastigotes and transgenic cells stably expressing the Cas9 and T7 RNA-polymerase were selected for 200 μg/mL hygromycin resistance.

### Generation of a LeishIF4E2(+/-) mutant by CRISPR-Cas9

To generate LeishIF4E2(+/-) mutants we used the two 5’ and 3’ sgRNAs designed to create double-strand breaks (DSBs) upstream and downstream of the LeishIF4E2 coding region and the LeishIF4E2 repair cassette fragment containing the G418 resistance marker. The three PCR products (4 μg of each) were transfected into mid-log phase transgenic cells expressing Cas9 and T7 RNA-polymerase and cells were further selected for resistance to 200 μg/mL G418 [25].

The sgRNA sequences used to delete the LeishIF4E2 gene were obtained from LeishGEdit.net [26]. The sgRNAs contained the highest-scoring 20 nt sequence within 105 bp upstream or downstream of the target gene. The sequences of the sgRNAs were blasted against the *L*. *mexicana* genome in TriTrypDB to verify that the sgRNAs were specific for LeishIF4E2 exclusively (E value = 0.001 and 8e^-5^). We also ran a BLAST analysis with the drug resistance repair cassette that contained the homology sequence to the UTR of LeishIF4E2 to specifically target the insertion of the selection marker. The repair cassette showed an E value of 5e^-9^ suggesting very high specificity of the system. The sgRNA target sequences and the homology arms on the repair cassette fully matched the target sequence of LeishIF4E2.

### PCR amplification of sgRNA templates

DNA fragments encoding LeishIF4E2 specific 5’ and 3’ guide RNAs for cleavage upstream and downstream to the LeishIF4E2 target gene were generated. All primers are listed in S1 Table. The template for this PCR reaction consisted of two fragments: one contained the common sgRNA scaffold fragment; the other contained a primer that included the T7 RNA polymerase promoter (small letters) fused to the gRNA (5’ or 3’), targeting LeishIF4E2 (capital letters) and a short sequence overlapping with the scaffold fragment (small letters, 5’gRNA (LmxM.19.1480) or 3’gRNA (LmxM.19.1480, S1 Table). Each of these two fragments (1 μM each) was annealed to the partially overlapping scaffold fragment and further amplified with two small primers (2 μM each) derived from the T7 promoter (G00F) and the common scaffold fragment (G00R, S1 Table). The reaction mixture consisted of dNTPs (0.2 mM), HiFi Polymerase (1 unit, Phusion, NEB) in GC buffer with MgCl_2_ (NEB), in a total volume of 50 μL. The PCR conditions included an initial denaturation at 98°C for 2 min, followed by 35 cycles of 98°C for 10 s, annealing at 60°C for 30 s and extension at 72°C for 15 s. All PCR products were gel-purified and heated at 94°C for 5 min before transfection.

### PCR amplification of the LeishIF4E2 replacement fragment

A DNA fragment designed to repair the DSB surrounding the LeishIF4E2 target gene was amplified by PCR. The LeishIF4E2 specific primers were derived from the 5’ and 3’ endogenous untranslated regions (UTR) sequences upstream and downstream to the LeishIF4E2 gene based on the LeishGEdit database (http://www.leishgedit.net/Home.html) and the sequences of the antibiotic repair cassette, The primers were Upstream Forward (LmxM.19.1480) and Downstream reverse (LmxM.19.1480, S1 Table). Capital letters represent the UTR sequences of the LeishIF4E2 gene and small letters represent the region on the pT plasmid that flanks the UTR adjacent to the antibiotic resistance gene. The PCR for generating the fragment used for repair of the DSBs on both sides of the gene targeted for deletion was performed using the pTNeo plasmid as a template. The resulting fragments promote the integration of the drug resistance marker by homologous recombination at the target site. The reaction mixture consisted of 2 μM of each primer, dNTPs (0.2 mM), the template pTNeo (30 ng), 3% (v/v) DMSO, HiFi Polymerase (1 U of Phusion, NEB) in GC buffer (with MgCl_2_ to a final concentration of 1.5 mM) in a total volume of 50 μL. PCR conditions included initial denaturation at 98°C for 4 min followed by 40 cycles of 98°C for 30 Seconds (s), annealing at 65°C for 30 s and extension at 72°C for 2 min 15 s. The final extension was performed for 7 min at 72°C. All PCR products were gel purified and heated at 94°C for 5 min before transfection.

### Diagnostic PCR to confirm the deletion of LeishIF4E2

Genomic DNA from the drug-resistant cells was isolated 14 days post transfection using DNeasy Blood & Tissue Kit (Qiagen) and analyzed for the presence of the LeishIF4E2 gene, using specific primers derived from the UTRs of LeishIF4E2. The primers used were LeishIF4E2 (5’UTR) forward (P1) and LeishIF4E2 (3’UTR) Reverse (P2). A parallel reaction was performed to detect the presence of the G418 resistance gene with primers derived from its ORF: G418 Forward (P3) and G418 Reverse G418 Reverse (P4). An additional reaction confirming the insertion of the G418 cassette at the target site was performed with primers G418 Forward P3, and LeishIF4E2 (3’UTR) Reverse P2. All primers are listed in S1 Table. Genomic DNA from Cas9/T7 *L*. *mexicana* cells was used to detect the presence of the LeishIF4E2 gene. The reaction mixture consisted of 2 μM of each primer, gDNA (100 ng), dNTPs (0.2 mM), HiFi Polymerase (1 U Phusion, NEB) in GC buffer with MgCl_2_ (NEB) in a total volume of 50 μl. PCR conditions included initial denaturation at 98°C for 4 min followed by 35 cycles of 98°C for 30 s, annealing at 60°C for 30 s and extension at 72°C for 2 min 15 s. Final extension was done for 7 min at 72°C. PCR products were separated on 1% agarose gels.

### Generation of LeishIF4E2 add-back parasites and SBP-tagged LeishIF4E2 expressers

The hemizygous mutant LeishIF4E2(+/-) cells were transfected with an episomal vector that restored full-length LeishIF4E2_1-281_ expression. The plasmid was derived from pTPuro [24] which confers resistance to puromycin. The LeishIF4E2 add-back gene from *L*. *mexicana* was tagged with the Streptavidin binding peptide (SBP, ~4 kDa), enabling its further identification in the transgenic parasites by antibodies against the SBP tag [13]. The tagged LeishIF4E2 was amplified with primers LeishIF4E2_1-281_ Forward and LeishIF4E2_1-281_ Reverse, with *Bam*HI and *Xba*I sites introduced at the 5’ ends of these primers (S1 Table). The *Bam*HI/*Xba*I cleaved PCR product of LeishIF4E2 was cloned into the *Bam*HI and *Xba*I sites of pTPuro, replacing the pre-existing LeishIF4E1-SBP that was already cloned in that plasmid between two intergenic regions derived from the HSP83 genomic locus. The resulting pTPuro-LeishIF4E2-SBP expression vector was transfected into the LeishIF4E2(+/-) mutant, and cells were selected for their resistance to puromycin (100 μg/mL).

To express the full-length LeishIF4E2-SBP_1-281_ and the C-terminal truncated protein LeishIF4E2-SBP_1-217_ in transgenic parasites, the two ORFs were cloned into the *Bam*HI/*Xba*I sites of the pX-based vector, designed to tag the proteins at their N-terminus between two intergenic regions derived from the HSP83 genomic locus (pX-H-N-SBP-H) [13]. The two resulting vectors, pX-H-N-SBP-LeishIF4E2_1-281_-H and pX-H-N-SBP LeishIF4E2_1-217_-H, were transfected into WT cells that were selected for resistance to G418 (200 μg/mL G418).

### Growth analysis

*L*. *mexicana* M379 WT, Cas9/T7 expressing cells, the LeishIF4E2(+/-) mutant, the LeishIF4E2 add-back, LeishIF4E2-SBP_1-281_ and LeishIF4E2-SBP_1-217_ cells were cultured as promastigotes at 25 ^o^C in M199 medium with supplements (above). Cells were seeded at a concentration of 5 X 10^5^ cells/mL, and counted daily during 5 consecutive days. The curves were obtained from three independent repeats.

### Western analysis

Cells in the mid-log phase of growth (10 mL) were harvested and washed twice with phosphate buffered saline (PBS). The cell pellet was resuspended in 300 μl of PBS supplemented with a 2 X cocktail of protease inhibitors (Sigma) and 4 mM iodoacetamide (Sigma) with (+) phosphatase inhibitors: 25 mM sodium fluoride, 55 mM β-glycerophosphate and 5 mM sodium orthovanadate. Cells were lysed by the addition of 65 μl of 5 X Laemmli sample buffer and heated at 95°C for 5 min. Cell extracts (40 μL) were resolved over 10% SDS-PAGE, blotted and probed using specific primary and secondary antibodies.

Antibodies against LeishIF4E2 (rabbit polyclonal, 1:2,000, Adar Biotech) and against the SBP tag (mouse monoclonal, 1:10,000, Millipore), were used to detect the endogenous and tagged LeishIF4E2 proteins, respectively. The blots were developed by incubation with specific peroxidase-labelled secondary antibodies against rabbit (KPL, 1:10,000 for LeishIF4E2) and mouse (KPL, 1:10,000 for SBP).

### Translation assay

Global translation levels were monitored using the non-radioactive SUnSET (Surface SEnsing of Translation) assay. This assay is based on the incorporation of puromycin, an amino-acyl tRNA analog, into the A site of translating ribosomes [27]. Cells were incubated with puromycin (1 μg/mL, Sigma) for 1 hr and then washed twice with PBS. Cell pellets were resuspended in 300 μl of PBS, denatured in Laemmli sample buffer and boiled for 5 min. Cells treated with cycloheximide (100 μg/mL) prior to the addition of puromycin served as a negative control. Samples were resolved over 10% SDS-Polyacrylamide gel electrophoresis (SDS-PAGE). The gels were blotted and subjected to western analysis using monoclonal mouse anti-puromycin antibodies (DSHB, 1:1,000) and secondary peroxidase labeled anti-mouse antibodies (KPL, 1:10,000).

### Phase contrast microscopy of *Leishmania* promastigotes

Late log-phase cells from different lines were harvested, washed in cold PBS, fixed in 2% paraformaldehyde in PBS and mounted on glass slides. Phase contrast microscope images were captured at X100 magnification with a Zeiss Axiovert 200M microscope equipped with an AxioCam HRm CCD camera.

### Flow cytometry analysis of *Leishmania*

Cell viability was verified by incubation of the cells with 20 μg/mL propidium iodide (PI) for 30 min. The stained cells were analyzed using the ImageStream X Mark II Imaging flow cytometer (Millipore) with an X60/0.9 objective. Data from channels representing bright field, and fluorescence (PI) emission at 488 nm (to evaluate cell viability) were recorded for 20,000 cells for each analyzed sample. IDEAS software [28] generated the quantitative measurements of the focused, single live cells for all four examined cell strains. Cell shape was quantified using circularity and length features applied to the bright field image processed by an Adaptive Erode mask. Representative scatter plots are shown for single cells and for circularity (cell shape). Recorded emission of the PI in the gated population evaluated cell viability.

### Measurements of flagellar length

Changes in the flagellar length for each cell were recorded using IDEAS software. A customized mask based on subtraction of the Adaptive Erode mask from the skeleton mask was created for measuring only the flagella without the cell soma. Based on this mask we created a length feature on the Brightfield Image and used it to compare flagella length between different samples (S2 Table).

### Data analysis

IDEAS software [28] was used to generate the quantitative measurements of images recorded for the examined cell population. The focus quality of each cell was first determined by measuring the gradient root mean square (RMS) value. The cells with high RMS value in the histogram were gated to select cells in focus. Next, single cell populations were gated from the scatter plot of aspect ratio/area to exclude cell aggregates; the intensity of PI staining was used to exclude dead cells. The remaining living single cells in focus were subjected to image analysis to determine cell morphology. To obtain cell shape a customized Adaptive Erode mask was used on the bright field channel, with a coefficient of 78. We further customized this mask to exclude the flagellum from the cell shape analysis, to measure, circularity and elongation features. A predetermined threshold value of 4 was set to define circularity. Elongation values represent the ratio between cell length and width. Representative scatter plots are presented for focused single cells and for circularity. All data shown are from a minimum of three biological replicates.

### *In vitro* macrophage infection assay

*L*. *mexicana* LeishIF4E2(+/-) mutants and add-back cells, WT and transgenic parasites expressing Cas9/T7, were seeded at a concentration of 5 X 10^5^ cells/ mL and allowed to grow for 5 days to reach stationary growth phase. WT, Cas9/T7 expressers, LeishIF4E2(+/-) and add-back cells were washed with DMEM (Dulbecco’s Modified Eagle Medium) and labeled by incubation with 10 μM carboxyfluorescein succinimidyl ester (CFSE) in DMEM at 25°C for 10 min. The cells were washed with DMEM, counted and used to infect RAW 264.7 macrophages. The macrophages (5 X 10^5^) were pre-seeded a day in advance in chambered slides (Ibidi) and incubated with the parasites at a ratio of 10:1 for 1 h in 300 μl medium. The cells were washed three times with PBS and once in DMEM to remove extracellular parasites. The infected macrophages were either fixed immediately for further analysis by confocal microscopy, or incubated for a further 24 h at 37°C in an atmosphere containing 5% CO_2_. The infected macrophages were then processed for confocal microscopy. A single representative section of Z-projections (maximum intensity) produced by Image J software is presented in all the figures. The infectivity values were determined using the cell counting plugin in ImageJ. We first counted the number of infected cells in a total of 100 macrophages, and then counted the number of internalized parasites within the infected cells, 1 or 24 hr following infection. Statistical analysis was performed using GraphPad Prism 5. We used the non-parametric Kruskal-Wallis test to determine significant differences in the infectivity and in the average number of parasites per infected macrophage.

### Confocal microscopy of *Leishmania* promastigotes

Infected macrophages, following 1 or 24 hr of infection were washed with PBS, fixed in 2% paraformaldehyde for 30 min, washed once with PBS and permeabilized with 0.1% Triton X-100 in PBS for 10 min. Nucleic acids were stained with 4′,6-diamidino-2-phenylindole (1 μg/mL DAPI, Sigma) and the cells were washed three times with PBS. The slides were observed using an inverted Zeiss LSM 880 Axio-observer Z1 confocal laser scanning microscope with Airyscan detector. Cells were visualized using Zeiss Plan-Apochromat oil objective lens of X 63 and numerical aperture of 1.4. Z-stacked images were acquired with a digital zoom of X 5.58 (X 1.8 for broad fields), using the Zen lite software (Carl Zeiss microscopy). Images were processed using Image J software package. A single representative section of the compiled Z-projections produced by Image J software is presented in all the figures.

### Mass spectrometry analysis

To characterize the proteomic differences between the LeishIF4E2(+/-) mutant and the Cas9/T7 control cells we performed mass spectrometry (MS) analysis of total cell lysates. Total cell lysates from mid log stage promastigotes of Cas9/T7 and LeishIF4E2(+/-) were resuspended in a buffer containing 100 mM Tris HCl pH 7.4, 10 mM DTT, 5% SDS, 2 mM iodoacetamide and a cocktail of protease inhibitors (Sigma). Cell lysates were precipitated using 10% trichloro-acetic acid (TCA) and the pellets were washed with 80% acetone. The mass spectrometric analysis was performed by the Smoler Proteomics Center at the Technion, Israel.

### Mass spectrometry (MS)

Proteins were reduced using 3 mM DTT (60°C for 30 min), followed by modification with 10 mM iodoacetamide in 100 mM ammonium bicarbonate for 30 min at room temperature. This was followed by overnight digestion in 10 mM ammonium bicarbonate in trypsin (Promega) at 37°C. Trypsin-digested peptides were desalted, dried, resuspended in 0.1% formic acid and resolved by reverse phase chromatography over a 30 min linear gradient with 5% to 35% acetonitrile and 0.1% formic acid in water, a 15 min gradient with 35% to 95% acetonitrile and 0.1% formic acid in water and a 15 min gradient at 95% acetonitrile and 0.1% formic acid in water at a flow rate of 0.15 μl/min. MS was performed using a Q-Exactive Plus Mass Spectrometer (Thermo) in positive mode set to conduct a repetitively full MS scan followed by high energy collision dissociation of the 10 dominant ions selected from the first MS scan. A mass tolerance of 10 ppm for precursor masses and 20 ppm for fragment ions was set.

### Statistical analysis for enriched proteins

Raw MS data were analyzed by the MaxQuant software, version 1.5.2.8 [29]. The data were searched against the annotated *L*. *mexicana* proteins from the TriTrypDB [30]. Protein identification was set at less than a 1% false discovery rate. The MaxQuant settings selected were a minimum of 1 razor/unique peptide for identification with a minimum peptide length of six amino acids and a maximum of two mis-cleavages. For protein quantification, summed peptide intensities were used. Missing intensities from the analyses were substituted with values close to baseline only if the values were present in the corresponding analyzed sample. The log_2_ of LFQ intensities [31] were compared between the three biological repeats of each group on the Perseus software platform [32], using a t-test. The enrichment threshold was set to a log_2_ fold change > 0.8 and p < 0.05. The annotated proteins were categorized manually and by GO annotation (below).

### Categorization of enriched proteins by the gene ontology (GO) annotation via TriTrypDB

Enriched proteins were classified by the GO Annotation tool in TriTrypDB, based on Biological Functions. The threshold for the calculated enrichment of proteins based on their GO terms was set at 3 fold, with a p <0.05. This threshold eliminated most of the general groups that represented parental GO terms. GO terms for which only a single protein was annotated were filtered out as well. In some cases, GO terms that were included in other functional terms are not shown leaving only the representative terms.

### Statistical analysis

Statistical analysis was performed using GraphPad Prism version 5. Each experiment was performed independently at least three times and the individual values were presented as dots. For experiments with a higher number of repeats results are expressed as Mean ± SD. Statistical significance was determined using Wilcoxon paired t-test for matched pairs test or Kruskal-Wallis with Dunn’s multiple comparison test for comparing three or more groups. Significant *P* values were marked with stars (*, **, ***) representing *P* <0.05, *P* <0.01 and *P* <0.001, respectively.

## Results

### LeishIF4E2 is a cytoplasmic protein with an extended C-terminal region

**Fig 1:**
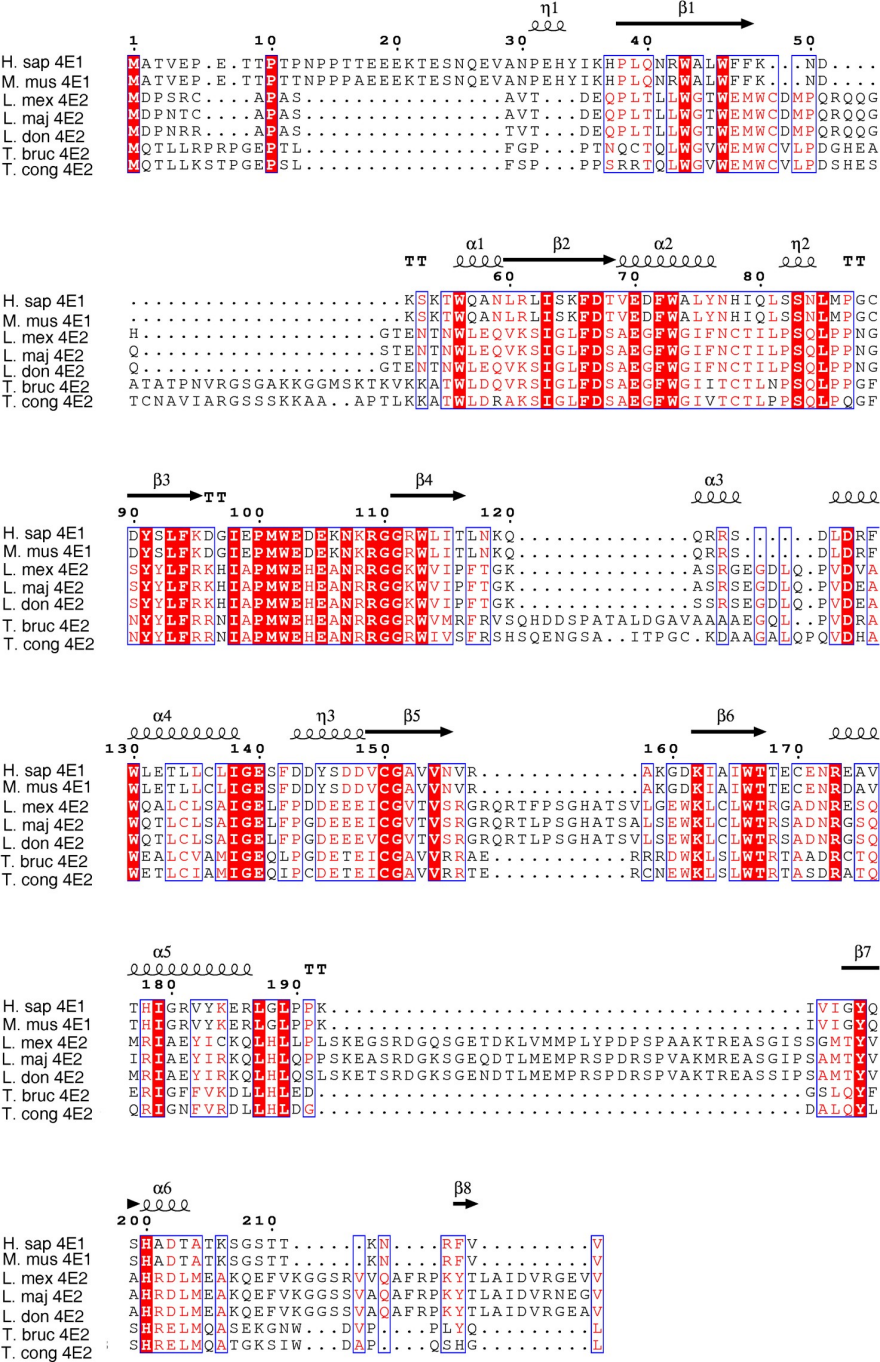
Multiple sequence alignment of the LeishIF4E2. **(A)**The ORFs of LeishIF4E2 from different *Leishmania* and *Trypanosoma* specis along with mammalian eIF4E1 were aligned using Jalview (2.10.5). The sequences were retrieved from *L*. *mexicana* (L. mex, LmxM.19.1480), *L*. *major* (L. maj, LmjF.19.1500); *L*. *donovani*, (L. don, LdBPK_191520.1), *T*. *brucei* (T. bruc, Tb927.10.16070) and *T*. *congolense* (T. cong, TcIL3000_0_03820). The alignment file was saved in FASTA format. The final alignment showing the predicted secondary structure of LeishIF4E2 was developed using the downloaded PDB file (5EHC, DOI: 10.2210/pdb5EHC/pdb) of *Homo sapiens* eIF4E1 (https://www.rcsb.org/), along with the FASTA alignment file, using the online ESPript 3 tool [60]. Secondary structure elements of the aligned sequences are (α: alpha helices, ƞ: 3_10_-helix, β: beta-strands, TT: strict β-turns). White letters over a red background indicate identical residues and red letters over a white background indicate sequence similarity.

Given the multiple eIF4E paralogs in *Leishmania*, and the assumption that they vary in their functional assignments, we examined the sequences of the different LeishIF4E paralogs in search for non-conserved gaps and extensions. We evaluated the homology between LeishIF4E2 and the mammalian eIF4E, as well as its homology with the other LeishIF4E paralogs. Fig 1 and S1A show that LeishIF4E2 has a non-conserved C-terminal extension whereas LeishIF4E3 and LeishIF4E4 contain extended N-terminal regions. These N-terminal extensions have been widely examined for phosphorylation sites [18,33,34] and structural features [35]. The different LeishIF4Es differ from their mammalian counterpart, showing only 29.4–40.4% similarity. LeishIF4E2 from different *Leishmania* and *Trypanosoma* species are also diverged from their mammalian ortholog (Human eIF4E) (S1B and S1C Fig) and show a sequence variability among themselves as well when compared to LeishIF4E1, with 26.4–30.9% similarity (S1D Fig).

In Fig 1, the ORFs of LeishIF4E2 from different *Leishmania* and *Trypanosoma* species were aligned using Jalview (2.10.5). The final alignment also demonstrates the secondary structure which was developed using the 3D structure of *Homo sapiens* eIF4E1 from PDB (https://www.rcsb.org/), along with the FASTA alignment file generated using the online ESPript 3 tool. Despite the partial sequence similarities, the structural core of the eIF4E orthologs (1–217,) is conserved (Fig 1 and [36]). The predicted secondary structure of LeishIF4E2 is composed of alpha helices, beta strands and beta turns. LeishIF4E2 from all *Leishmania* species have an extended C-terminal region which is absent from the trypanosome orthologs (*T*. *brucei* and *T*. *congolensi* in Fig 1). The *Leishmania* C-terminus appears to be highly disordered as predicted by the DISOPRED2 server through the XtalPred site [37]. There are four adjacent proline residues after amino acid 217 which are known to break secondary structures. These appear within the extended C-terminus (S1A Fig).

We examined the possibility that the disordered region at the C-terminus is subject to proteolytic cleavage both *in vivo* and *in vitro*. For monitoring cleavage *in vivo*, we generated *Leishmania* lines expressing the full length LeishIF4E2_1-281_ and the shorter version devoid of the C-terminal extension (LeishIF4E2_1-217_), both tagged with SBP at their N-terminus, so that the protein could be detected with anti-SBP antibodies irrespective of C-terminal cleavage (S2A and S2B Fig). The transgenic cell lines expressing full length LeishIF4E2 (1–281) and the shorter version LeishIF4E2_1-217_ were validated by western analysis using antibodies specific for the SBP tag (S2A Fig). Western blots of the total proteins versus the supernatant of disrupted cells (S2B Fig), show a reduced reaction with anti-SBP antibodies in both lines. However, the full-length protein (LeishIF4E2_1-281_) exhibits partial degradation (S2B Fig) a feature not observed for the shorter version of LIF4E2_1-217_. This reduction is compatible with protein cleavage, especially given the appearance of a smaller band in the western blots of the full-length protein. Tagging the protein at its N-terminus enabled us to visualize the bands that could represent LeishIF4E2 partially degraded from its C-terminus. LeishIF4E2_1-217_ does not show any degradation like the full-length protein (LeishIF4E2_1-281_), supporting our interpretation that degradation occurs at the C-terminus.

In addition to *in vivo* expressed LeishIF4E2, we monitored the potential cleavage of the disordered C-terminus *in vitro*. The full length recombinant LeishIF4E2, with an N-terminal histidine tag, was expressed in *E*. *coli* and affinity purified over a nickel column. The eluted fractions were examined by western analysis using an antibody against histidine. The affinity purified protein migrated as a major band of >25 kDa, and a minor higher band of <35 kDa. (S2C Fig, Size differences between the *in vivo* and *in vitro* assays could be due to the different tags fused to LeishIF4E2 or to post translational modifications in the different hosts). The appearance of the 25 kDa band supports our *in vivo* observation of the inherent instability of the C-terminal disordered region.

We further investigated the localization of LeishIF4E2 within *Leishmania* cells by examining transgenic parasite cells expressing the SBP-tagged LeishIF4E2 by immunohistochemical confocal microscopy. Mid-log cells (1.2 x 10^7^ cells/mL) were harvested, washed and fixed with paraformaldehyde. The fixed cells were incubated with the primary anti-SBP antibody followed by a secondary anti-mouse antibody labeled with a fluorophore (Alexa Fluor 488 green). Nuclear and kinetoplast DNA were stained with DAPI. S3 Fig shows that LeishIF4E2 is non-uniformly distributed occurring mostly in the cytoplasm.

### Deletion of a single copy of LeishIF4E2 by CRISPR-Cas9

LeishIF4E2 is the least studied cap-binding protein paralog and we therefore attempted to delete its two alleles and examine how this deletion affects the parasite phenotype. Specific sgRNAs that targeted LeishIF4E2 at its 5’ and 3’ UTRs were transfected into the *L*. *mexicana* Cas9/T7 expressing line [19, 24]. The sgRNAs were designed to cleave around the target gene promoting its replacement with the G418 or Blasticidin repair fragments. The LeishIF4E2 deletion cell line was selected in the presence of G418 (200 μg/mL) and a diagnostic PCR analysis indicated that a single allele was eliminated. Attempts to delete the other copy of LIF4E2 with the Blasticidin replacement cassette did not result in viable cells.

**Fig 2:**
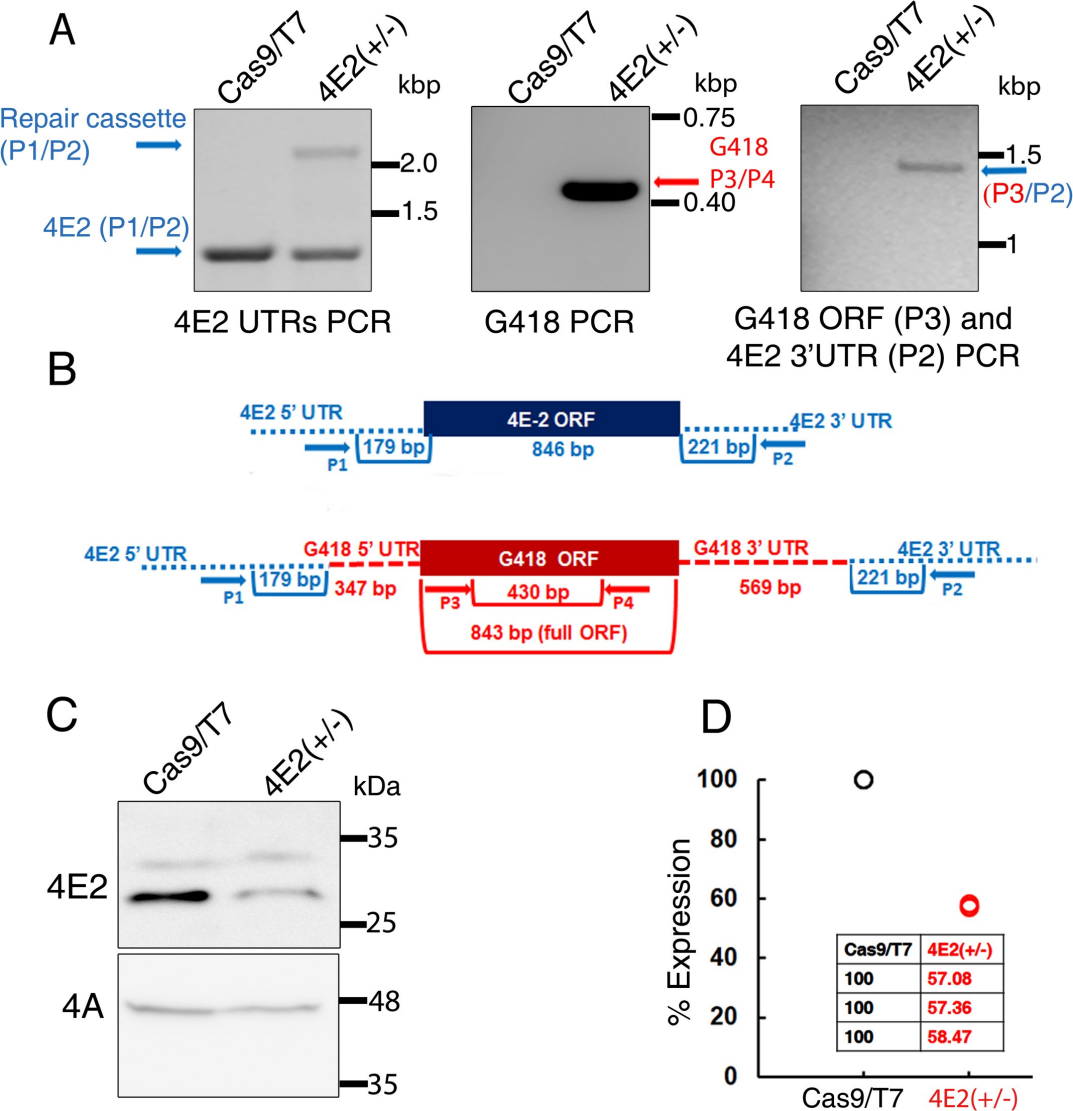
CRISPR-Cas9 mediated hemizygous deletion of LeishIF4E2. **(A)** Diagnostic PCR was performed to confirm the deletion of single allele of LeishIF4E2. Genomic DNA was extracted from *L*. *mexicana* Cas9/T7 cells and from the LeishIF4E2(+/-) mutant. PCR was performed using different combinations of primers derived from the LeishIF4E2 5’ UTR (Forward) and 3’ UTR (Reverse) (P1/P2 –left panel); G418 ORF (Forward) and Reverse (P3/P4, middle panel); and a primer set derived from the G418 resistance gene, forward and the 3’ UTR (Reverse) (P3/P2). **(B)** Schematic representation of LeishIF4E2 locus and primers (represented by arrows). The PCR was applied to test the presence or absence of the LeishIF4E2 gene and the G418 resistance marker. Primers derived from the LeishIF4E2 UTRs are shown in blue and primers derived from the ORF of G418 are shown in red. **(C)** Western analysis monitoring the protein level of LeishIF4E2 in the LeishIF4E2(+/-) mutant and in Cas9/T7 control was performed using LeishIF4E2 specific antibodies. LeishIF4A-1 served as a loading control. **(D)** Dot plot representing the densitometry analysis of the LeishIF4E2 protein levels in the LeishIF4E2(+/-) mutant and in the Cas9/T7 controls.

Diagnostic PCR was performed with genomic DNA of the mutant using several primer pairs. Primers derived from the 5’ and 3’ UTRs of the LeishIF4E2 transcript were used to monitor the presence of the LeishIF4E2 gene (primers P1/P2, Fig 2A, left panel). The reaction generated two products: a 1.2 Kb product representing the endogenous LeishIF4E2 gene, and a second product of ~2.1 Kb corresponding to the integrated G418 resistance gene flanked by the 5’ and 3’ LeishIF4E2 UTRs that replaced the LeishIF4E2 gene. A similar PCR control reaction using the Cas9/T7 control gDNA as a template yielded only a single product (~1.2 Kb), representing the endogenous LeishIF4E2 gene (Fig 2A left panel). The presence of the G418 selection marker in the genome was verified by primers P3/P4 that were derived from the G418 gene and gave a 450 bp product only in the mutant of LeishIF4E2 and not in the Cas9/T7 control (Fig 2A middle panel). To validate that the G418 selection marker replaced the LeishIF4E2 gene, the integration site was amplified using primers P2/P3, P3 was derived from the G418 ORF and P2 was derived from the 3’ UTR of LeishIF4E2. This PCR product (1.4 kb) was observed only in the mutant and not in the Cas9/T7 control DNA (Fig 2A right panel). Fig 2B shows the positions of different primers used for the diagnosis PCR. The different PCR reactions confirmed the deletion of a single copy of the LeishIF4E2 gene from the genome of *Leishmania* cells expressing Cas9/T7.

The effect of the LeishIF4E2 hemizygous deletion on target protein expression was examined by western analysis of cell extracts obtained from the LeishIF4E2(+/-) mutant, as compared to control cells expressing Cas9/T7. The blots were reacted with LeishIF4E2 specific antibodies (Fig 2C upper panel). Antibodies against LeishIF4A served as a loading control (Fig 2C lower panel). Fig 2D shows a densitometry analysis of the LeishIF4E2 expression in the deletion mutant as compared to the Cas9/T7 control. The LeishIF4E2(+/-) mutant shows a 40% reduction in the protein levels as compared to control Cas9/T7 cells. We observed two bands in the western analysis using antibodies specific for LeishIF4E2, one at ~31 kDa and another above 25 kDa. A pattern that could fit protein cleavage was also observed with the recombinant LeishIF4E2 tagged at its N-terminus (S2C Fig) most probably due to cleavage at the C-terminus.

Expression of LeishIF4E2 was recovered in an add-back line. The LeishIF4E2(+/-) cells were transfected with a pTPuro-derived plasmid that contained the SBP-LeishIF4E2 gene flanked by HSP83 intergenic regions. The cells were selected for their resistance to puromycin, and expression of the newly introduced gene was verified by western analysis using antibodies against the SBP-tag (S4 Fig).

### The LeishIF4E2(+/-) mutant shows altered promastigote morphology

**Fig 3:**
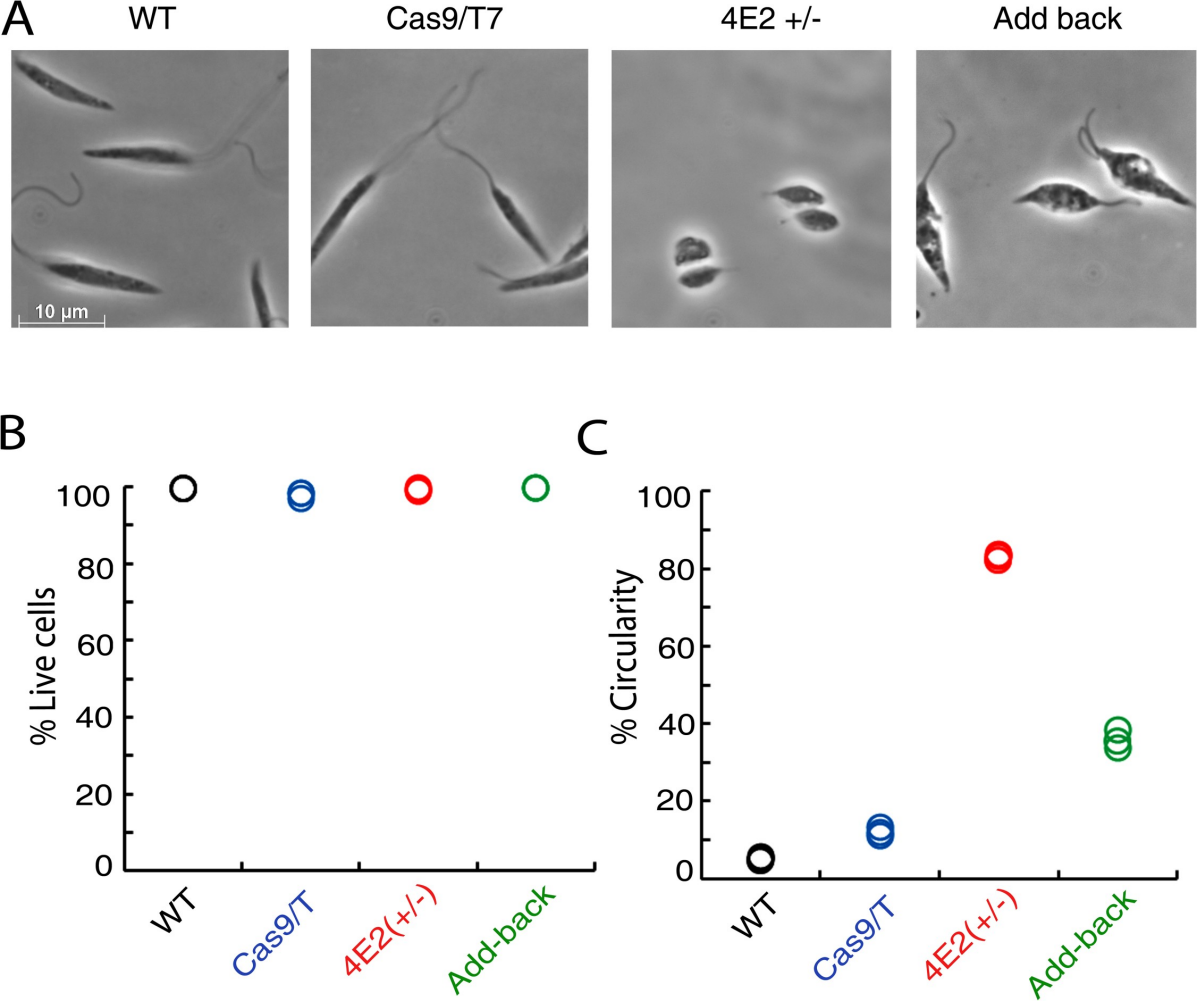
The LeishIF4E2(+/-) mutant shows altered promastigote morphology. **(A)** Mid-log phase (Day 2) promastigotes of WT, Cas9/T7 expressers, LeishIF4E2(+/-) cells, and LeishIF4E2 add-back cells were fixed with 2% paraformaldehyde and visualized by phase contrast microscopy at 100x magnification. WT, Cas9/T7 expressing cells showed normal promastigote morphology while LeishIF4E2(+/-) were round with reduced flagellar length. **(B)** All the cell lines were stained with 20 μg/mL propidium iodide (PI) for 30 min and cell viability was analyzed using the ImageStream X Mark II Imaging flow cytometer (Millipore). 20,000 cells were analyzed for each sample and percent viable cells were determined. **(C)**. The circularity of single, viable and focused cells from each of the cell lines was quantified using flow cytometry, and is shown as percentage of the total number of cells measured. Data from three independent experiments are shown.

Mid log phase (Day 2) promastigotes of LeishIF4E2(+/-), Cas9/T7 expressers, WT cells, and LeishIF4E2 add-back parasites were grown at 25 ^o^, washed with PBS and fixed with 2% paraformaldehyde. The slides were visualized by phase contrast microscopy at 100 X magnification. The LeishIF4E2(+/-) mutant showed a defective morphology; the cells had become round, flagellum length was reduced and deviated from the typical promastigote form. Control WT and Cas9/T7 expressing cells exhibited normal promastigote features with a typical elongated shape and a long protruding flagellum. Normal promastigote morphology of elongated cells and flagellum growth were restored when expression of LeishIF4E2(+/-) was recovered in the add-back cells by episomal transfection of SBP tagged-LeishIF4E2 with pT-Puro-H-LeishIF4E2_1-281_-SBP-H. Restoration of LeishIF4E2 expression led to recovery of promastigote-like cell morphology and flagellum growth nearly similar to control WT and Cas9/T7 cells (Fig 3A right panel and S5 Fig for the broad field, S6 and S7 Figs).

We further used flow cytometry imaging to analyze the cell shape and viability of the cell populations as previously described [28, 38] (S6 Fig). The circularity parameter is calculated for each cell as the average radius divided by the radial variance. Typical round cells exhibit low radial variance, whereas ruffled or elongated cells have high variance of radii that results in lower circularity parameter. Cells were gated on the basis of being viable, single (non-aggregated) and focused (S6A and S6B Fig), with 20,000 cells from each group analyzed. We measured cell viability by incubating with Propidium Iodide (PI) for 30 min. The viability of the LeishIF4E2(+/-) mutant was similar to that of control cells and cell viability was comparable between all the groups tested (Fig 3B and S6A). In parallel we analyzed and quantified the changes in cell shape. In the LeishIF4E2(+/-) mutant, ~83% cells were deviated from their normal promastigote morphology being round and with a reduced and shortened flagellum. In control WT and Cas9/T7 expressing cells, only 5.14% and 11.8% were round, respectively. As expression of LeishIF4E2 was restored in the add-back cells, we noticed a recovery from their mutant phenotype, with only 35% round cells (Fig 3C and S6C Fig).

Changes in the flagellar length for each cell were also recorded using the acquisition software that was then analyzed by the IDEAS software (S7 Fig). We created the custom mask based on subtraction of the Adaptive Erode mask from the skeleton mask, thus measuring only the flagella without the cell soma. Based on this custom mask we created a length feature on the Brightfield Image and used it to compare flagella length between different samples. In WT and CAS9/T7 expressers we could identify a flagellum in 91.8–93.1% of focused cells compared with only 40.6% of the LeishIF4E2(+/-) mutants. S2 Table shows that 81.2% of the add-back cells had restored flagella. For cells in which we could identify a flagellum there was a substantial difference in the flagellum length between the LeishIF4E2(+/-) (5.50 micrometer) and the WT or Cas9/T7 control cells (12.72 and 10.19 micrometers, respectively). The flagellar length of the add-back cells recovered and attained 9.93 micrometer S2 Table.

### Global translation and growth are not affected by the deletion of a single LeishIF4E2 allele

To investigate how the reduced level of LeishIF4E2 expression in the hemizygous mutant affected overall translation we used the SUnSET assay which is based on the incorporation of puromycin into the growing polypeptide chains. Puromycin is a structural analogue of amino acyl tRNA that occupies the ribosomal A site. Its integration into the polypeptide chains blocks their elongation and results in translation termination. Mid-log LeishIF4E2(+/-) mutant, control WT and Cas9/T7 expressing cells, LeishIF4E2-SBP_1-281_ and LeishIF4E2-SBP_1-217_ transgenic cells were incubated with puromycin (1μg/mL) for 1 hr. The cells were then harvested, extracted and resolved on SDS-PAGE. Puromycin incorporation was monitored by western analysis using anti-puromycin antibodies. A cycloheximide treated sample served as a negative control with no active puromycin incorporation.

**Fig 4:**
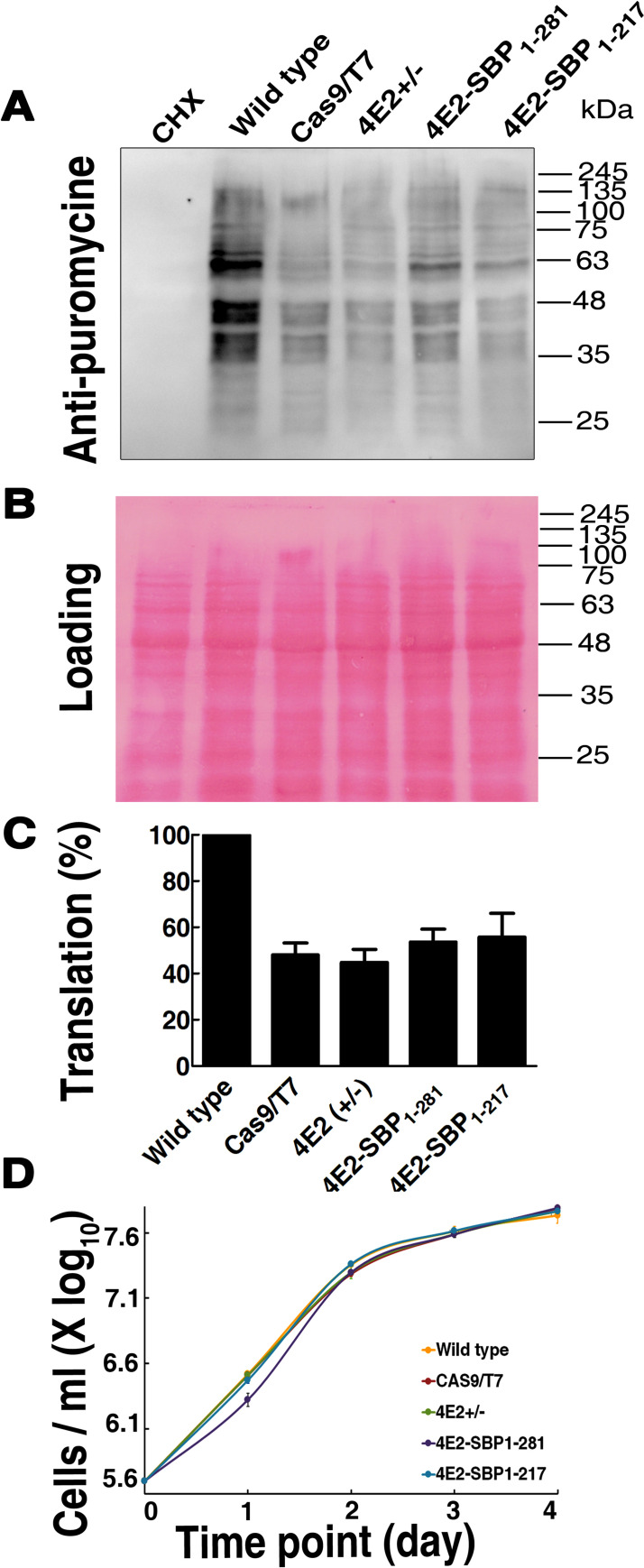
Global translation and growth are not altered in the LeishIF4E2(+/-) mutant cells. **(A)** LeishIF4E2(+/-) cells, WT, Cas9/T7 expressing cells and transgenic parasites expressing the full length LeishIF4E2_1-281_ and its truncated form devoid of the C-terminus, LeishIF4E2_1-217_ were incubated with 1 μg/mL puromycin for 1 hr. Cycloheximide treated cells were used as a negative control for complete inhibition of translation. Puromycin treated cells were lysed and resolved over 12% SDS-PAGE and subjected to western analysis using antibodies against puromycin. **(B)** Ponceau staining was used to indicate comparable protein loads. **(C)** Densitometry analysis of puromycin incorporation in the different cells lines, was compared to WT cells (considered as 100%). Data from all three independent experiments are represented. **(D)** All cells were cultured at 25 ^o^C in M199 containing essential nutrients. Cell counts were monitored daily during 4 consecutive days. The curves were obtained from three independent assays, error bars are also marked. Growth curves of WT cells are shown in yellow, Cas9/T7 are in brown, the LeishIF4E2(+/-) mutant cells are in green, cells expressing the full length LeishIF4E2 _1–281_ are in purple, and the truncated LeishIF4E2_1-217_ cells are in blue.

Global translation in the LeishIF4E2(+/-) mutants was hardly affected by the hemizygous deletion as compared to control Cas9/T7 expressing cells (Fig 4A). Densitometry analysis was normalized to the total protein load (Fig 4B) and showed that translation levels in Cas9/T7 and LeishIF4E2(+/-) mutants were 48% and 44.7%, respectively, as compared to WT (100%, Fig 4C). Translation of the transgenic parasites expressing the full length and truncated proteins (1–281 and 1–217, respectively) did not alter their translation efficiency of 53.6 and 55.7%, respectively. Reduced translation in the control Cas9/T7 as compared to WT has been previously reported [15] and could be due to the competition over the use of the translation machinery between the overexpressed and endogenous proteins. S8 Fig demonstrates that the global translation in cells expressing the non-related Chloramphenicol acetyltransferase (CAT) reporter was reduced (68.8%).

We further monitored the growth rates of the LeishIF4E2(+/-) mutant, control WT and Cas9/T7 expressing cells, LeishIF4E2-SBP_1-281_ and LeishIF4E2-SBP_1-217_ transgenic cells. All lines were cultured at 25 ^o^C in M199 containing essential nutrients, seeded at an initial concentration of 4 X 10^5^ cells/mL and counted on a daily basis during four consecutive days. The growth curves (Fig 4D) showed no difference between the proliferation of the LeishIF4E2(+/-) mutant cells and control lines (WT and Cas9/T7) or the transgenic lines expressing LeishIF4E2_1-281_ or LeishIF4E2_1-217_. This observation was in line with the results obtained in the SUnSET assay demonstrating that global translation in the LeishIF4E2(+/-) mutant cells was the same as in control cells. Overexpression of the two versions of LeishIF4E2 did not affect global translation indicating that the presumable C-terminal cleavage of LeishIF4E2 does not interfere with translation. Although additional analysis of translated transcripts is required, such a profile could be explained if LeishIF4E2 was involved in regulation of specific transcripts rather than global translation *per sei*.

### The LeishIF4E2(+/-) mutant cells show reduced infectivity in cultured macrophages

**Fig 5:**
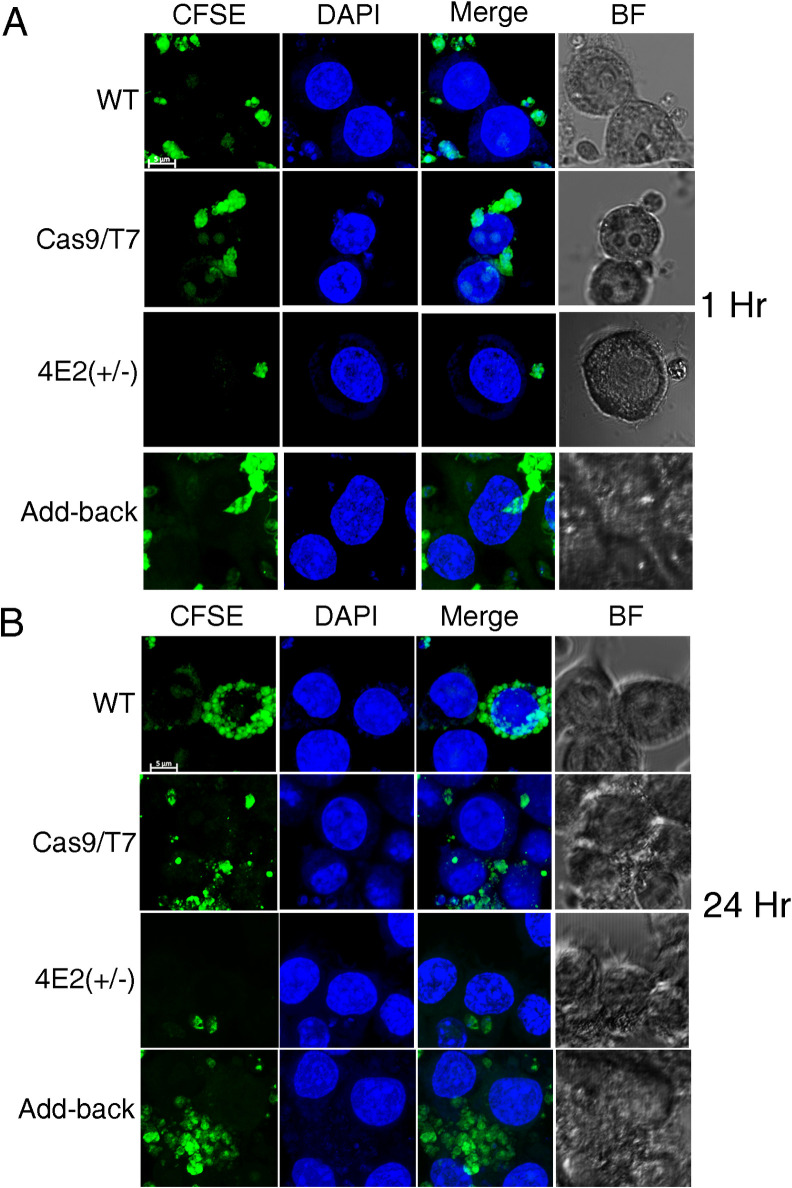
The LeishIF4E2(+/-) mutant cells show reduced infectivity to macrophages. Stationary phase (Day 5) *L*. *mexicana* WT, Cas9/T7 expressing cells, LeishIF4E2(+/-) mutant cells and the add-back LeishIF4E2 line were pre-stained with CFSE (green), counted, washed and used to infected RAW 264.7 macrophages, at a ratio of 10:1 for one hour. The cells were then washed to remove unattached parasites, and the macrophages were cultured for 1h **(A)** or 24h **(B)** at 37°C. Macrophage nuclei were stained with DAPI and the infected macrophage slides were processed for confocal microscopy, showing a Z-projection produced by Image J software. Fields containing 100 cells were further evaluated to quantify the infection. The scale bar represents 5 μm.

Since the LeishIF4E2(+/-) mutant cells showed a defective morphology having short flagella and round body structure, we examined their ability to infect cultured murine macrophages using the RAW 267.4 line. *Leishmania* parasites from respective cell lines were pre-stained with carboxyfluorescein succinimidyl ester (CFSE), and used to infect the macrophages at a multiplicity of 10:1 parasites per macrophage. Infection lasted for 1 hr at 37°C. The macrophages were washed to remove unbound parasites and the cells were fixed with paraformaldehyde (2%) and processed for confocal analysis. DAPI staining was used to visualize the large macrophage nuclei. The infected macrophage cultures were examined by confocal microscopy either immediately (1 hr), or 24 hr post infection. These two time points allowed us to keep track of the initial entry of *Leishmania* and their subsequent multiplication inside the host macrophages. Infectivity of the LeishIF4E2(+/-) mutant cells was compared to that of WT and control Cas9/T7 expressing cells. We counted 100 macrophages from different fields to determine the infectivity index. The results, shown in Figs 5A and 5B, S9 Fig (for a broad field view), S10A and S10B Figs, indicate that infectivity of the LeishIF4E2(+/-) mutant was impaired, as observed at both 1 hr and 24 hrs post infection. Quantitation of the infection fields (S9 and S10 Figs and S3 Table) showed that the LeishIF4E2 mutant cells were able to infect only 48.5% of the macrophages after 1hr, with an average of 2.2 parasites per cell whereas WT and Cas9/T7 controls infected 87.8 and 91.5% of the cells, with 2.8 and 2.2 parasites per cell, respectively. The infectivity of the add-back cells was restored with 89.4% of the cells infected after 1 hr with an average of 3 parasites per macrophage (Figs 5A and 5B and S9 and S3 Table).

A more pronounced difference was observed after 24 hr of infection. WT and Cas9/T7 controls infected 95–99% of the macrophages with 5.4 and 5 parasites per cell respectively, whereas the LeishIF4E2(+/-) mutant infected only 65% of the macrophages, with an average number of 1.8 parasites per cell, indicating that they did not multiply within the macrophages as in the control cells. The add-back parasites recovered their ability to infect the macrophages (97.8%) with an average of 4.8 parasites per cell showing that they recovered their ability to multiply within the macrophages. Thus, the original impaired infectivity of the mutant LIF4E2(+/-) was due to the reduced expression of the protein and once expression was restored infectivity recovered (Figs 5, S9 and S10 and S3 Table). Parasites per macrophage are represented after normalization to 1 hr uptake. This was achieved by dividing parasites per macrophages at the 24 hr time point with parasites per macrophages at 1 hr time point (S3 Table).

### Proteomic analysis of the LeishIF4E2(+/-) mutant cells shows up- and down-regulation of proteins involved in specific cellular processes

**Fig 6:**
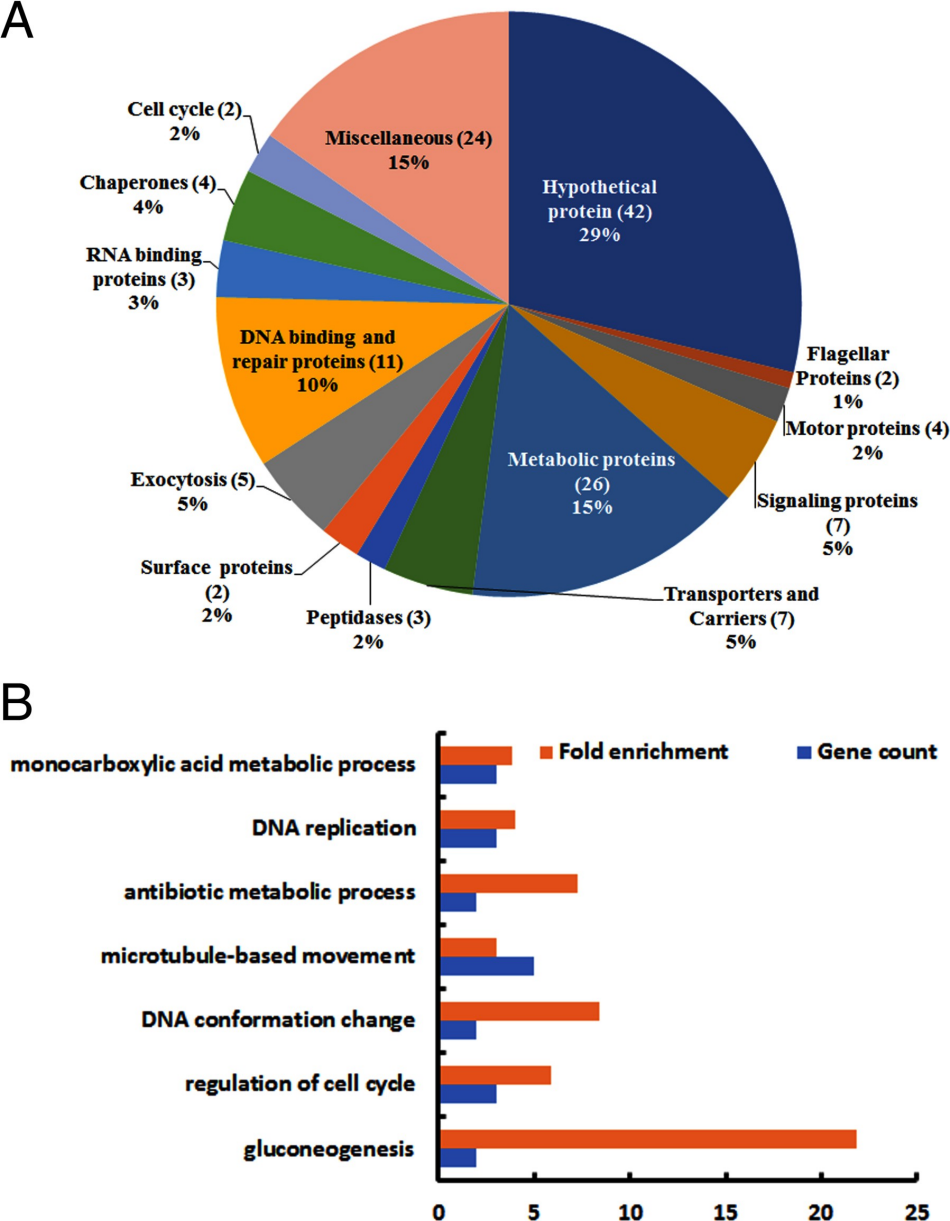
The categorized proteome of the upregulated proteins in LeishIF4E2(+/-) mutant cells as compared to Cas9/T7 control cells. The proteomic content of LeishIF4E2(+/-) and Cas9/T7 cells was determined by LC-MS/MS analysis, in triplicates. Raw mass spectrometric data were analyzed and quantified using the MaxQuant software and the peptide data were searched against the annotated *L*. *mexicana* proteins listed in TriTrypDB. The summed intensities of the peptides that served to identify the individual proteins were used to quantify changes in the proteomic content of specific proteins. Statistical analysis was done using the Perseus software. Proteins that were upregulated in the LeishIF4E2(+/-) mutant by 1.7 fold as compared to Cas9/T7 cell extracts, with p<0.05 are shown. **(A**) Proteins in LeishIF4E2(+/-) that were upregulated (>1.7 fold) as compared to Cas9/T7 extracts were clustered manually into functional categories. The pie chart represents the summed intensities of upregulated proteins in each category, in the LeishIF4E2(+/-) mutant. Numbers in brackets indicate the number of proteins in each category, % represent their summed relative intensity in the analysis. **(B)** Enriched proteins were classified by the GO enrichment tool in TriTrypDB, based on Biological Function. The threshold for the calculated enrichment of proteins based on their GO terms was set for 2.5 fold, with p<0.05. This threshold eliminated most of the general groups that represented parental GO terms. GO terms for which only a single protein was annotated were filtered out as well.

To examine potential differences in the proteomic profile of LeishIF4E2(+/-) mutant we performed a MS analysis of the total cell extracts of the LeishIF4E2(+/-) mutant cells, as compared to Cas9/T7 control cells. Three independent cultures of mid-log cells were used for the proteome analysis and all samples were analyzed in the same run. The *L*. *mexicana* genome in TritrypDB was used to assign the peptides to their source proteins which were later quantified by the MaxQuant software. The proteomic comparison identified proteins that were increased relative to the Cas9/T7 control proteome (Fig 6) or decreased (S4 Table), using a threshold change of at least 1.7 fold, with p <0.05. Perseus software platform was used for the statistical analysis ([32] and S4 Table). The statistical analysis showed that 142 proteins were upregulated and 95 proteins were downregulated in LeishIF4E2(+/-) compared with Cas9/T7 control cells.

Fig 6A and S4 Table describe the manually categorized groups of the up- and down-regulated proteins compared to Cas9/T7 control cells. The upregulated proteins related to various cellular processes involved in cell metabolism, DNA repair and replication, signaling and microtubule-based movement. The amastin-like surface antigen was also upregulated; this protein family is known to increase in amastigotes. Signaling proteins that included various kinases and phosphatases were also upregulated.

The upregulated proteins were also evaluated by the Gene Ontology (GO) enrichment analysis through the TriTrypDB platform based on their biological functions. Enrichment threshold was set at 3 fold. Fig 6B and S4 Table highlight the GO enrichment of the upregulated protein groups, each containing at least 2 proteins. In line with the manually categorized proteins, the GO enrichment analysis also show that the upregulated proteins relate to general cellular and DNA metabolism and to microtubule-based movement. We note a strong increase in proteins involved in gluconeogenesis, which are typical of amastigotes.

The manually categorized proteins that were downregulated in the LeishIF4E2(+/) mutant as compared to the Cas9/T7 control cells (S4 Table) mostly related to the flagellar rod, the cytoskeleton dyneins and a few ribosomal proteins (L22, L18, S15 and S10). Other downregulated proteins in the LeishIF4E2(+/-) proteome included signaling and metabolic proteins. A number of proteins known to be involved in parasite virulence [39–42] such as surface proteins like GP46/PSA and hydrophilic acetylated surface proteins, cysteine and metallo-peptidases were also reduced. The GO enrichment analysis for the downregulated genes highlighted cellular amino acid biosynthetic processes and cyclic nucleotide biosynthetic processes, both know to decrease in amastigotes.

### The LeishIF4E2(+/-) morphology resembles axenic amastigotes

**Fig 7:**
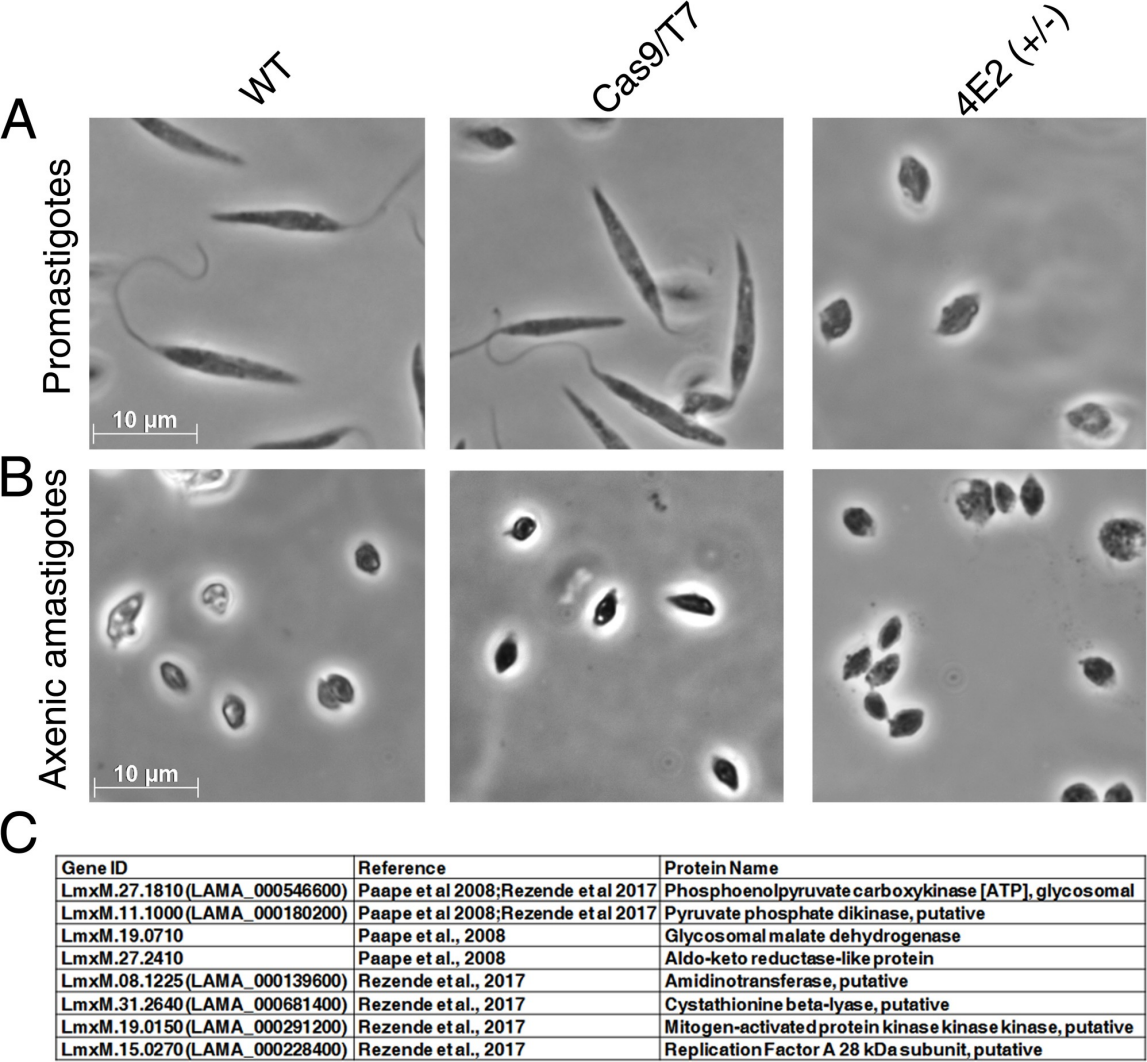
LeishIF4E2(+/-) mutant cells easily transform to axenic amastigote-like cells. **(A)** Promastigotes of the LeishIF4E2(+/-) mutant, control WT, and Cas9/T7 expressing cells grown under normal conditions are shown. **(B)** Morphology of cells transferred to conditions that induce differentiation to axenic-amastigotes (33°C/pH 5.5) during four days. Images were captured at 100x magnification with a Zeiss Axiovert 200M microscope equipped with AxioCam HRm CCD camera. The scale bar is 10 μm. **(C)** Shared upregulated proteins in the mutant LeishIF4E2(+/-) promastigotes (compared to Cas9/T7 cells) and in published amastigotes proteome. The total protein of the LeishIF4E2(+/-) mutant promastigotes was compared to the proteome of Cas9/T7 cells. The list of upregulated proteins was further compared with the proteins enriched in the amastigote proteome of the virulent *L*. *amazonensis* PH8 strain (de Rezende et al, PLOS NTD 2017) and of *L*. *mexicana* amastigotes (Paape et al, Mol Cell Prot 2008). *L amazonensis* gene IDs were converted to *L*. *mexicana*, for the sake of comparison.

The LeishIF4E2(+/-) mutant promastigotes were small and round and had a very short flagellum (Figs 7A and S7). This morphology resembles the changes observed during differentiation to axenic-like amastigotes under normal growth conditions. We therefore examined how the mutant cells responded to conditions known to generate axenic amastigotes. L. *mexicana* LeishIF4E2(+/-) promastigotes along with control WT and Cas9/T7 cells were grown to late log phase and transferred to conditions that induce differentiation to axenic amastigotes *in vitro* (pH 5.5, 33°C) for four days. The differentiated LeishIF4E2(+/-) cells maintained a similar morphology, except that they became smaller, resembling axenic amastigote structure (Fig 7B).

We compared the repertoire of proteins that were enriched in the LeishIF4E2(+/-) mutant promastigotes with the published proteome specific to amastigotes [43,44]. This comparison highlighted eight overlaps between the proteins that were upregulated in the LeishIF4E2(+/-) mutant and in amastigotes (Fig 7C and S5 Table), as reported previously [45,46]. The shared proteins included enzymes required for gluconeogenesis, such as the phosphoenolpyruvate carboxykinase and pyruvate phosphate dikinase. These two enzymes are key players in the gluconeogenesis pathways which are active in the amastigote stage of *Leishmania* and are required for the intracellular survival of the parasite within mammalian host cells [47]. We also noticed that the aldo-keto reductase was upregulated in LeishIF4E2(+/-). Aldo-keto reductase is suggested to have a role in the removal of the ketoaldehyde metabolites derived from lipids and trioses [46]. Other proteins upregulated in LeishIF4E2(+/-) included cystathionine beta-lyase (methionine biosynthesis) and signaling kinases like mitogen-activated protein kinase. Enzymes involved in lipid metabolism pathways were shown to increase in *Leishmania* amastigotes, as these change their energy source on entering mammalian macrophages [48]. Fewer overlaps were observed for down-regulated proteins. These included the peptidyl-prolyl cis-trans isomerase, putative, a protein implicated in protein folding [49]. Downregulation of a poly zinc finger protein 2 of unknown function was also shared.

## Discussion

The current study aims to understand one of the least studied *Leishmania* cap-binding paralogs, LeishIF4E2, using CRISPR-Cas9 mediated gene knock-out. We successfully deleted one of the two LeishIF4E2 alleles and studied the effect of this deletion on various cellular processes, including growth, morphology, metabolism, infectivity to macrophages, and overall proteome.

LeishIF4E2 does not associate with any eIF4G partner and co-migrates with polysomes on sucrose gradients [10], however, this profile does not indicate its biological role. The inability to bind an LeishIF4G partner is similar to the translation repressor 4E-HP [8], however, the absence of LIF4G binding does not necessarily indicate that the protein is a repressor. We recently showed that LeishIF4E1, which also does not pair with any LeishIF4G does not appear to function as a translation repressor [15].

The ortholog of LeishIF4E2 from *T*. *brucei*, TbEIF4E2, associates with a stem-loop binding protein that binds to histone mRNA, but its role in translation activation or repression remained elusive. Here, generation of a hemizygous mutant of LeishIF4E2 by CRISPR-Cas9 gene knockout enables us to explore the potential role of LeishIF4E2 in translation in *Leishmania* and how this deletion affects the proteomic profile. Our attempts to delete both alleles of LeishIF4E2 were unsuccessful, although we were able to obtain a null mutation of another cap-binding protein, LeishIF4E1, by the same approach [15] supporting that LeishIF4E2 performs an essential function. Although the deletion of the single allele of LeishIF4E2 reduced its expression, it did not affect global translation and growth rates of the mutant cells. However, the hemizygous mutant had an altered proteome profile. MS analysis and GO enrichment analysis demonstrated that the protein families with decreased expression are categorized as flagellar rod and cytoskeletal proteins; this is compatible with the observed defects in LeishIF4E2(+/-) mutant cellular morphology. On the other hand, proteins involved in cytoskeletal movement were upregulated, conferring a minimal movement capacity to the round mutant cells. The fact that global translation appears unaffected may be due to a downstream effect. If LeishIF4E2 is responsible for transcript specific translation, only specific subsets of genes would be affected, and this would not be reflected in global translation assays. A downstream protein involved in protein stability could also affect the changes in the proteomic profile of the mutant cells.

The LeishIF4E2 sequences are highly conserved among the *Leishmania* species and less with trypanosomes. LeishIF4E2 possesses an extended C-terminal domain predicted to be disordered. This extended C-terminus is absent from all other LeishIF4Es emphasizing that it is unique to LeishIF4E2. It is also absent from the *T*. *brucei* and *T*. *congolensi* orthologs of LeishIF4E2 making this C-terminus is specific to the *Leishmania*. We find that the disordered C-terminus is subject to proteolytic cleavage, both *in vivo* and *in vitro*. In cell extracts antibodies against the SBP tag could recognize LeishIF4E2 tagged at its N-terminus. These antibodies recognized both the full-length protein (LeishIF4E2_1-281_) and its C-terminal truncated product (LeishIF4E2_1-217_) when LeishIF4E2 was tagged at its N-terminus. In LeishIF4E2_1-281_, cleavage of the C-terminus generated the shorter version of the protein that could be identified by the antibodies to the N-terminal tag. A similar approach using recombinant LeishIF4E2 purified from bacterial extracts showed both the presumable full-length and cleaved protein lacking the disordered C-terminus. Here too antibodies against the His tag interacted with the recombinant LeishIF4E2 only when it was tagged at its N-terminus. Since we did not observe any difference in total translation or growth rates between cell lines expressing the full length or the shorter version the biological role of this cleavage and its products remains elusive.

Complete or partial deletion of other LeishIF4E paralogs has resulted in similar effects on cell morphology giving rise to mutant cells that were small, non-flagellated and impaired in their ability to infect macrophages. We note that this was also the case with the null mutant of LeishIF4E1 [15] and the hemizygous mutant of LeishIF4E3 [19]. We show here that the hemizygous deletion of LeishIF4E2 generates morphologically defective cells. In all these cases, we observed a similar pattern of behavior, namely that perturbation of individual LeishIF4Es results in altered morphology, defects in cytoskeletal motility proteins, flagellar growth and impaired infectivity. The different LeishIF4Es may fulfill each other’s role to a certain extent, allowing viability, but they may be responsible for translation of different sets of transcripts, since we expect that many genes are required to maintain a proper cytoskeleton and a functional flagellum. Impaired expression of discrete transcript groups could result in impaired shape and motility in the different mutants with different transcripts being affected. Based on previous reports in the field it is also expected that the flagellum is required for the entry of promastigotes into macrophages after transmission to the mammalian host, [50–53]. Apart from the defective morphology, the downregulation of important virulence factors such as discrete surface proteins, promastigote surface antigens (PSA/GP46), could result in the impaired infectivity we observed. Expression of the GP46/PSA surface antigen was previously shown to be involved in parasite virulence by protecting the parasite from complement mediated lysis [42]. We note that despite the morphological changes and the relative changes in the proteome, promastigote viability was not affected as compared to control cells, indicating that inhibition of cell motility, or changes in cell size do not necessarily affect cell viability. Analysis of the proteomic changes in the LeishIF4E2(+/-) mutant shows that along with the reduced expression of specific proteins, we observed an upregulation of proteins involved in DNA replication and repair, that could contribute to cell proliferation of the mutant cells, as compared to the controls. These could assist in maintain cell proliferation.

Genome wide tethering screens for identifying regulatory proteins that affect gene expression in *T*. *brucei* suggested that TbEIF4E2 acts as a translation repressor [54]. From our study we cannot conclude that LeishIF4E2 is a general translation repressor, since the translation assays monitoring *de novo* translation in the LeishIF4E2(+/-) mutant cells did not demonstrate any increase in protein synthesis activity or even cell growth. In addition, parasites expressing SBP tagged LeishIF4E2 did not show any decrease in global translation, ruling out the general translation repressive ability of LeishIF4E2. However, as indicated above, if LeishIF4E2 could function in a transcript-specific manner, it could possibly repress translation of specific transcripts without affecting global translation. Alternatively, LeishIF4E2 could serve as a transcript-specific translation factor that a reduction in its expression could lead to impaired translation of specific transcripts. Both alternatives rule out a role for LeishIF4E2 as a global translation repressor. We also cannot exclude the option that reduced expression of LeishIF4E2 had an indirect effect on the proteomic profile, through modulation of a small number of downstream proteins involved in translation. Contrasting roles for a specific cap-binding protein have been reported for the 4E-HP ortholog in higher eukaryotes, as 4E-HP fails to bind any eIF4G partner, and functions as a translation repressor during embryogenesis [55]. However, it also functions as a translation inducing factor for a specific set of transcripts translated under conditions of hypoxia [56].

We further considered the possibility that LeishIF4E2 is associated with stage differentiation. Our former publication [13] indicated that LeishIF4E2 expression was almost undetectable in axenic amastigotes. Expression of other cap-binding proteins was also reduced with only LeishIF4E1 maintaining its expression level at both life stages. LeishIF4E4 changed its modification profile, and LeishIF4E3 was reduced in axenic amastigotes. The complete absence of LeishIF4E2 expression in axenic amastigotes could indicate that stage transformation requires its suppression during differentiation. Indeed, we noticed that LeishIF4E2(+/-) mutants are easily differentiated into axenic amastigotes. We now see that reduction of LeishIF4E2(+/-) expression lead to upregulation of important enzymes involved in gluconeogenesis, as compared to Cas9/T7 expressers. This group of proteins was also reported to increase in amastigotes [43, 57]. Another protein that was upregulated when expression of LeishIF4E2 was reduced is the mitogen-activated protein kinase-kinase (LmxM.19.0150), a protein reported to increase in axenic amastigotes [57]. The *T*. *brucei* ortholog, TbEIF4E2, was shown to bind the stem-loop binding protein 2, SLBP2, a protein that associates specifically with histone mRNAs. In higher eukaryotes SLBP associates with polyribosomes as a result of continued synthesis and transport of the histone mRNP to the cytoplasm [58]. LeishIF4E2 is the only cap-binding protein that clearly associates with polysomal fractions that were separated over sucrose gradients; the other LeishIF4Es tested co-migrated with lower MW fractions of the sucrose gradient [10]. The repertoire of upregulated genes in the LeishIF4E2 (+/-) mutant shows enrichment of DNA repair and DNA binding proteins, some of which are also involved in DNA replication. Therefore, LeishIF4E2 could be directly or indirectly involved in maintaining DNA integrity and structure. However there is a need to do further investigation on this aspect. For comparison, proteins that relate to these groups were not upregulated in the LeishIF4E1(-/-) null mutant cells [15] suggesting unique roles for the different cap-binding proteins.

This study further emphasizes the complex nature of the network that regulates translation in trypanosomatids with potentially overlapping functions of the different cap-binding proteins. This is further emphasized when one considers the involvement of additional 4E-interacting proteins, such as Leish4E-IP1 [13] and Leish4E-IP2 [59] in modulating protein synthesis in this group of fascinating organisms that diverged early in the evolution of eukaryotes. The presence of multiple cap-binding proteins in this unicellular organism is intriguing as it appears that recruitment of this important group of proteins to perform specific functions is part of the successful machinery of adaptation to the changing environments that the parasites experience during their complex life cycle, transiting between insect vector and mammalian host.

## Supporting information

S1 Fig A Sequence alignment of the cap-binding protein paralogs of *Leishmania mexicana* indicating the N- and C-termini extensions. **(A)** Sequences were retrieved from *L*. *mexicana* parasites annotated in TriTrypDB. Alignment was generated using Jalview (2.10.5). The aligned sequences were derived from *L*.*mexicana* genome sequences from TritrypDB. The sequences used were LeishIF4E1 (4E1, LmxM.27.1620); LeishIF4E2, (4E2, LmxM.19.1480); LeishIF4E3 (4E3, LmxM.28.2500); LeishIF4E4 (4E4, LmxM.29.0450); LeishIF4E5 (4E5, LmxM.36.0590); LeishIF4E6 (4E6, LmxM.26.0240). N-terminal extensions are observed in LeishIF4E3 and LeishIF4E4, as previously reported. A C-terminal extension is observed only in LeishIF4E2. (**B)** The table shows percent similarities between the different *Leishmania* LeishIF4Es and the *Mus musculus* eIF4E. **(C)** The tables show the percent similarities between the different trypanosomatid orthologs of LeishIF4E2 with the *Homo sapiens* eIF4E. **(D)** The table shows % similarities between the different LeishIF4Es, and LeishIF4E1. Percent similarities were generated by EMBOSS needle (https://www.ebi.ac.uk/Tools/psa/emboss_needle/).
(TIF)

S2 Fig LeishIF4E2 is susceptible to C-terminal cleavage. *L*. *mexicana* cells expressing the N-terminally tagged full length SBP-LeishIF4E2_1-281_, the truncated version of LeishIF4E2_1-217_ and WT cells, were grown under normal conditions. **(A)** Cells were rapidly lysed in SDS-PAGE gel loading buffer, showing the total extracts. The blot was developed with antibodies against the SBP tag. **(B)** Lanes marked as Total were obtained from rapid lysis as in (A), and lanes marked as Supernatant were obtained from cell lysis with Triton X-100 incubated on ice for 10 min, followed by centrifugation to remove the insoluble fractions of the cell. The blot was developed with antibodies against the SBP tag. **(C)** Bacterial cells expressing the recombinant LeishIF4E2_1-281_ tagged with Histidine at its N-terminus were disrupted in a French Press, and the protein was affinity purified over a nickel column. The blot was developed using antibodies against the His tag.
(TIF)

S3 Fig LeishIF4E2 is localized in the cytoplasm. *L*. *amazonensis* cells expressing LeishIF4E2-SBP were grown under normal conditions. The cells were washed, fixed in paraformaldehyde and further processed for confocal microscopy. LeishIF4E2 was detected using monoclonal anti-SBP primary antibody and a secondary goat anti-mouse fluorescent antibody labeled with a green fluorophore (Alexafluore, 488 nM). The nuclear and kinetoplast DNA was stained using DAPI (blue). Images were taken using confocal microscopy showing a Z-projection that was produced by the Image J software. Scale bar: 10 μm. The digital zoom in **(A)** is 5.5 and in **(B)** is 1.8, giving a broader field.
(TIF)

S4 Fig Confirmation of add-back LeishIF4E2 expression. **(A)** Cell lysates of *L*. *mexicana* LeishIF4E2 add-back and WT cells were resolved over 10% SDS-PAGE followed by western analysis with antibodies directed against the SBP tag. **(B)** Ponceau staining of the blot served as a loading control.
(TIF)

S5 Fig Morphological changes of LeishIF4E2(+/-) and their recovery in the add-back cells (broad field). The mutant LeishIF4E2(+/-) mutant, the add-back cells along with WT and Cas9/T7 expresser cells were grown under normal conditions. The cells were fixed, and images were captured at X100 magnification with a Zeiss Axiovert 200M microscope equipped with AxioCam HRm CCD camera.
(TIF)

S6 Fig Flow cytometry for viability, gating of focused single cell population and cell shape quantification. *L*. *mexicana* WT, Cas9/T7 expressing control cells, LeishIF4E2(+/-) mutant and add-back promastigotes were subjected to Flow cytometry analysis. **(A)** Cell viability is represented for focused, single gated cells for all the different cell lines **(B)** Scatter plots representing gated focused single cell populations for different cell lines. **(C)** Cell shapes are being represented in terms of circularity or elongatedness as scatter plots for gated cell population.
(TIF)

S7 Fig Shortening of the flagella in the LeishIF4E2(+/-) hemizygous mutant and its recovery in the add-back cells. Data were acquired for WT, Cas9/T7, 4E2(+/-) and add-back cells in the assay using ImageStreamX mkII, Objective 60X/0.9NA. (**A)** An assay containing ~15,000 cells shows the percentage of cells with identifiable flagella (>0 um) as a dot plot. **(B)** The mean flagellum length of ~15,000 cells is shown as a dot plot. **(C)** Shows the normalized frequency of flagellar length (**D)** Representative Brightfield images of cells with various flagella length. Red lines show the mask used to identify the flagellum, numbers in dark blue show flagella length in micrometer for individual images.
(TIF)

S8 Fig Global translation in cells expressing the CAT reporter is reduced as compared to WT. **(A)** WT and transgenic cells expressing the CAT reporter (iCATi, I represents the HSP83 intergenic region that provides RNA processing signals) were incubated with 1 μg/mL puromycin for 1 hr. Cycloheximide treated cells were used as a negative control for complete inhibition of translation. Puromycin treated cells were lysed and resolved over 12% SDS-PAGE and subjected to western analysis using antibodies against puromycin. **(B)** Ponceau staining was used to indicate comparable protein loads. **(C)** Densitometry analysis of puromycin incorporation in the iCATi expressing cell line was compared to wild type (WT) cells (100%). Data from all three independent experiments are represented.
(TIF)

S9 Fig The LeishIF4E2(+/-) mutant cells show reduced infectivity to cultured macrophages (broad field). Stationary phase *L*. *mexicana* LeishIF4E2(+/-) mutant, WT, Cas9/T7 expressers and add-back cells, were pre-stained with the CFSE dye and used to infect RAW 264.7 macrophages at a ratio of 10:1 for one hour. The cells were then washed to remove excess parasites, and the macrophages were cultured for 1 hr (A) or 24 hr (B) post infection at 37°C. Macrophage nuclei were stained with DAPI and the infected macrophages were processed for confocal microscopy. A representative section of Z-projections (maximum intensity) produced by Image J software is shown. Fields of 200 cells were further evaluated to quantify the infection.
(TIF)

S10 Fig Statistical analysis of LeishIF4E2(+/-) mutant infectivity compared to controls. Parasite infectivity of cultured RAW 264.7 macrophages was estimated *in vitro* using Image J software. **(A)** The percentage of infected macrophages was determined by counting a total of 100 macrophages from three independent experiments**. (B)** The average number of parasites per infected cell is shown. Kruskal Wallis test in GraphPad Prism was used to determine the percentage of infected cells and for calculation of the average parasites per cell along with standard deviation values (SD). The percentage of infected macrophages (%) and the average number of parasites per macrophage in the LeishIF4E2(+/-) mutant were compared with each of the control lines: WT, Cas9/T7 expressing cells, the LeishIF4E2(+/-) mutant and the LeishIF4E2 add-back cells. *P* value < 0.001 is represented by ***, P value < 0.01 by ** and P value < 0.05 by *. The data for 1hr and 24 hr macrophage infections are shown in separate panels.
(TIF)

S1 Table List of primers. (XLSX)

S2 Table Measurements of flagellar length in the different parasite lines. Changes in the flagellar length for each cell taken by the acquisition software were calculated using IDEAS software. A customized mask was created for measuring only the flagella without the cell soma. Based on this mask, a length feature on the Brightfield Image was created and used to compare flagellum length between different samples, shown as A and B for the 1 and 24 hr infections.
(XLSX)

S3 Table Reduced infectivity of the LeishIF4E2(+/-) mutant cells as compared to WT and Cas9/T7 controls and to add-back cells. Parasite infectivity of cultured RAW 264.7 macrophages was estimated *in vitro*, using the Image J software. The percentage of infected macrophages was determined by counting a total of 100 macrophages from three independent experiments. The average number of parasites per infected cell is shown.
(XLSX)

S4 Table List of up- and down-regulated proteins in total extracts of the LeishIF4E2(+/-) mutant as compared to Cas9/T7 control cells. Raw MS data were analyzed and quantified using the MaxQuant software and the peptide data were searched against the annotated *L*. *mexicana* proteins listed in TriTrypDB. The proteomic content of 4E2(+/-) and Cas9/T7 cells was determined by LC-MS/MS analysis, in triplicates.
(XLSX)

S5 Table Comparison of the LeishIF4E2(+/-) proteome with published amastigote proteomes. The proteome of upregulated LeishIF4E2(+/-) mutant promastigotes was compared with the proteins enriched in the amastigote proteome of virulent *L*. *amazonensis* PH8 strain, as compared to the less virulent LV79 [44]. The up- and down-regulated proteins in LeishIF4E2(+/-) mutant promastigotes were also compared with the *L*. *mexicana* amastigote proteome [43].
(XLSX)
