# Supplementary figures and images for: Distinct features of the Leishmania cap-binding protein LeishIF4E2 revealed by CRISPR-Cas9 mediated hemizygous deletion
Source: PLoS Negl Trop Dis. 2021 Mar 24;15(3):e0008352. doi: 10.1371/journal.pntd.0008352 (PMC8021392; doi:10.1371/journal.pntd.0008352)

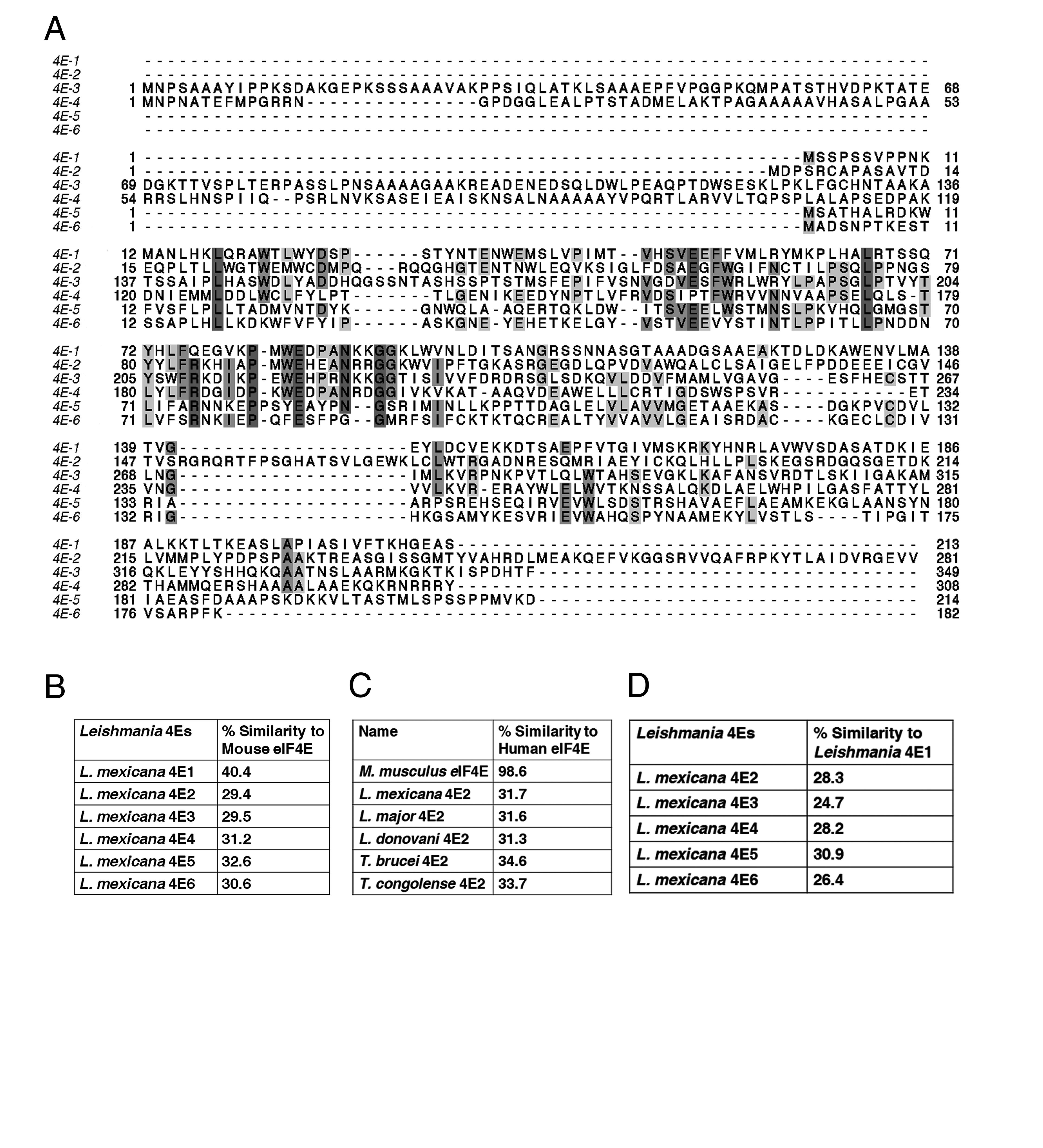

Supplement: S1 Fig — (A) Sequences were retrieved from L. mexicana parasites annotated in TriTrypDB. Alignment was generated using Jalview (2.10.5). The aligned sequences were derived from L.mexicana genome sequences from TritrypDB. The sequences used were LeishIF4E1 (4E1, LmxM.27.1620); LeishIF4E2, (4E2, LmxM.19.1480); LeishIF4E3 (4E3, LmxM.28.2500); LeishIF4E4 (4E4, LmxM.29.0450); LeishIF4E5 (4E5, LmxM.36.0590); LeishIF4E6 (4E6, LmxM.26.0240). N-terminal extensions are observed in LeishIF4E3 and LeishIF4E4, as previously reported. A C-terminal extension is observed only in LeishIF4E2. (B) The table shows percent similarities between the different Leishmania LeishIF4Es and the Mus musculus eIF4E. (C) The tables show the percent similarities between the different trypanosomatid orthologs of LeishIF4E2 with the Homo sapiens eIF4E. (D) The table shows % similarities between the different LeishIF4Es, and LeishIF4E1. Percent similarities were generated by EMBOSS needle (https://www.ebi.ac.uk/Tools/psa/emboss_needle/). (TIF) [file pntd.0008352.s001.tif]

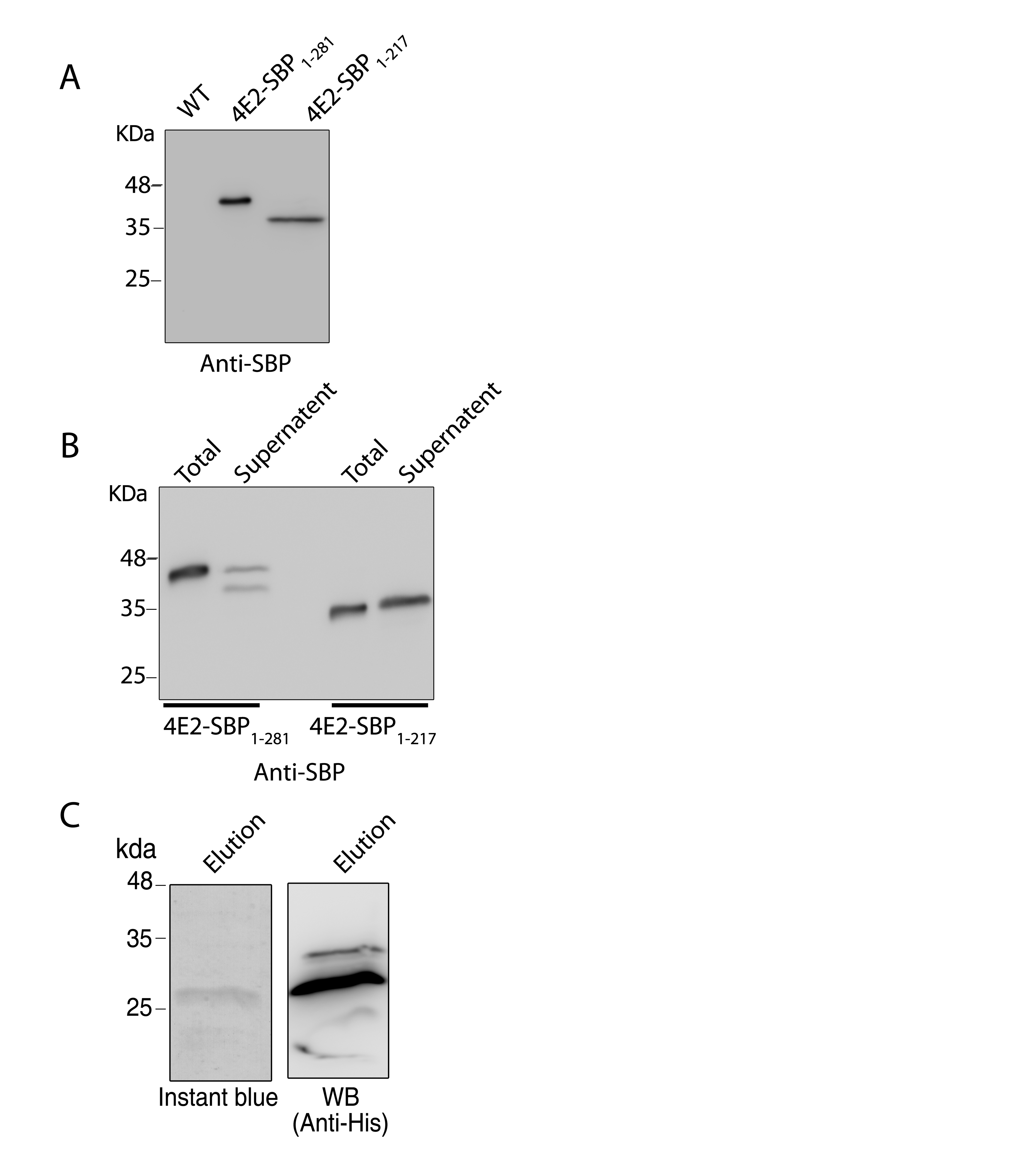

Supplement: S2 Fig — L. mexicana cells expressing the N-terminally tagged full length SBP-LeishIF4E21-281, the truncated version of LeishIF4E21-217 and WT cells, were grown under normal conditions. (A) Cells were rapidly lysed in SDS-PAGE gel loading buffer, showing the total extracts. The blot was developed with antibodies against the SBP tag. (B) Lanes marked as Total were obtained from rapid lysis as in (A), and lanes marked as Supernatant were obtained from cell lysis with Triton X-100 incubated on ice for 10 min, followed by centrifugation to remove the insoluble fractions of the cell. The blot was developed with antibodies against the SBP tag. (C) Bacterial cells expressing the recombinant LeishIF4E21-281 tagged with Histidine at its N-terminus were disrupted in a French Press, and the protein was affinity purified over a nickel column. The blot was developed using antibodies against the His tag. (TIF) [file pntd.0008352.s002.tif]

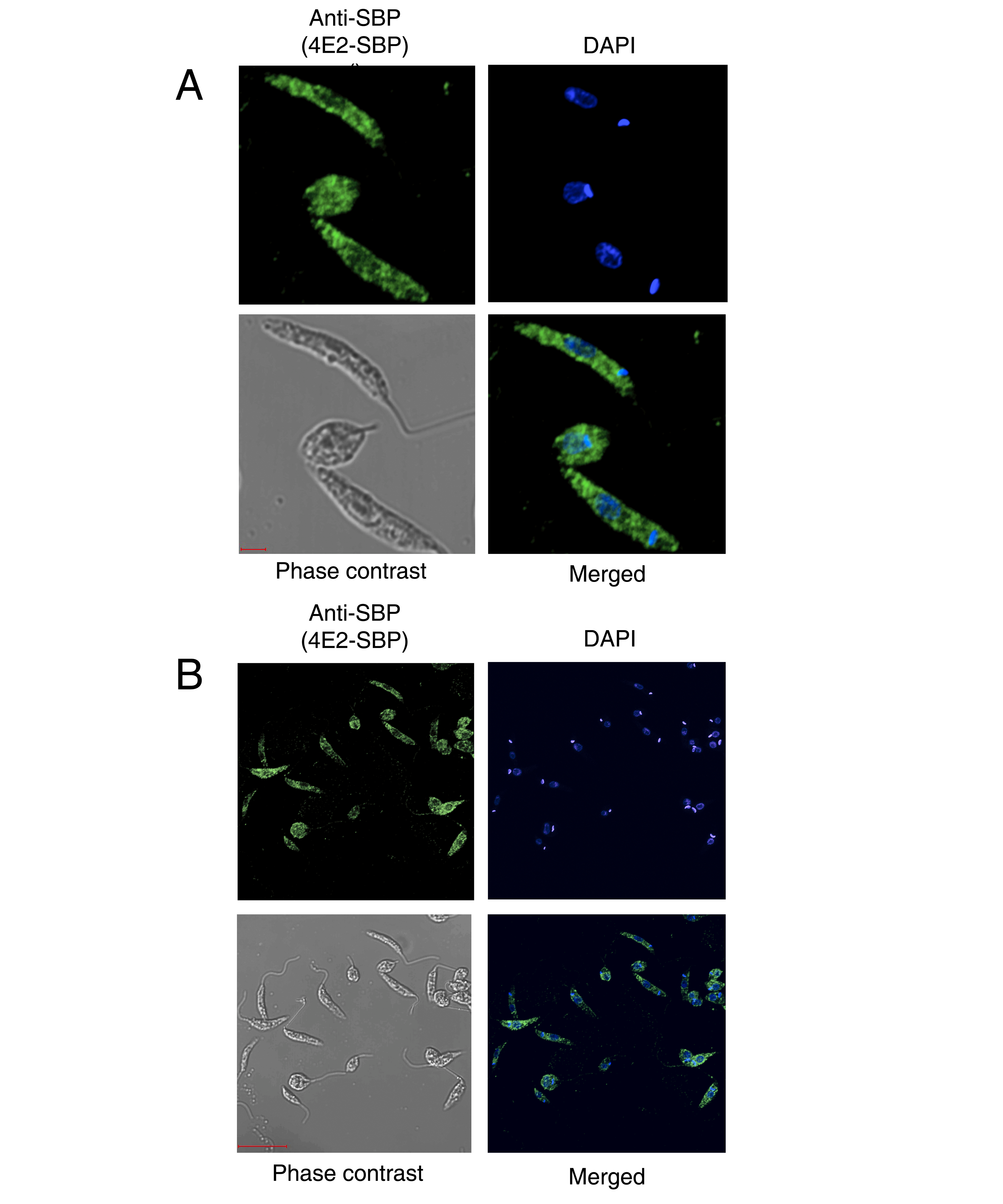

Supplement: S3 Fig — L. amazonensis cells expressing LeishIF4E2-SBP were grown under normal conditions. The cells were washed, fixed in paraformaldehyde and further processed for confocal microscopy. LeishIF4E2 was detected using monoclonal anti-SBP primary antibody and a secondary goat anti-mouse fluorescent antibody labeled with a green fluorophore (Alexafluore, 488 nM). The nuclear and kinetoplast DNA was stained using DAPI (blue). Images were taken using confocal microscopy showing a Z-projection that was produced by the Image J software. Scale bar: 10 μm. The digital zoom in (A) is 5.5 and in (B) is 1.8, giving a broader field. (TIF) [file pntd.0008352.s003.tif]

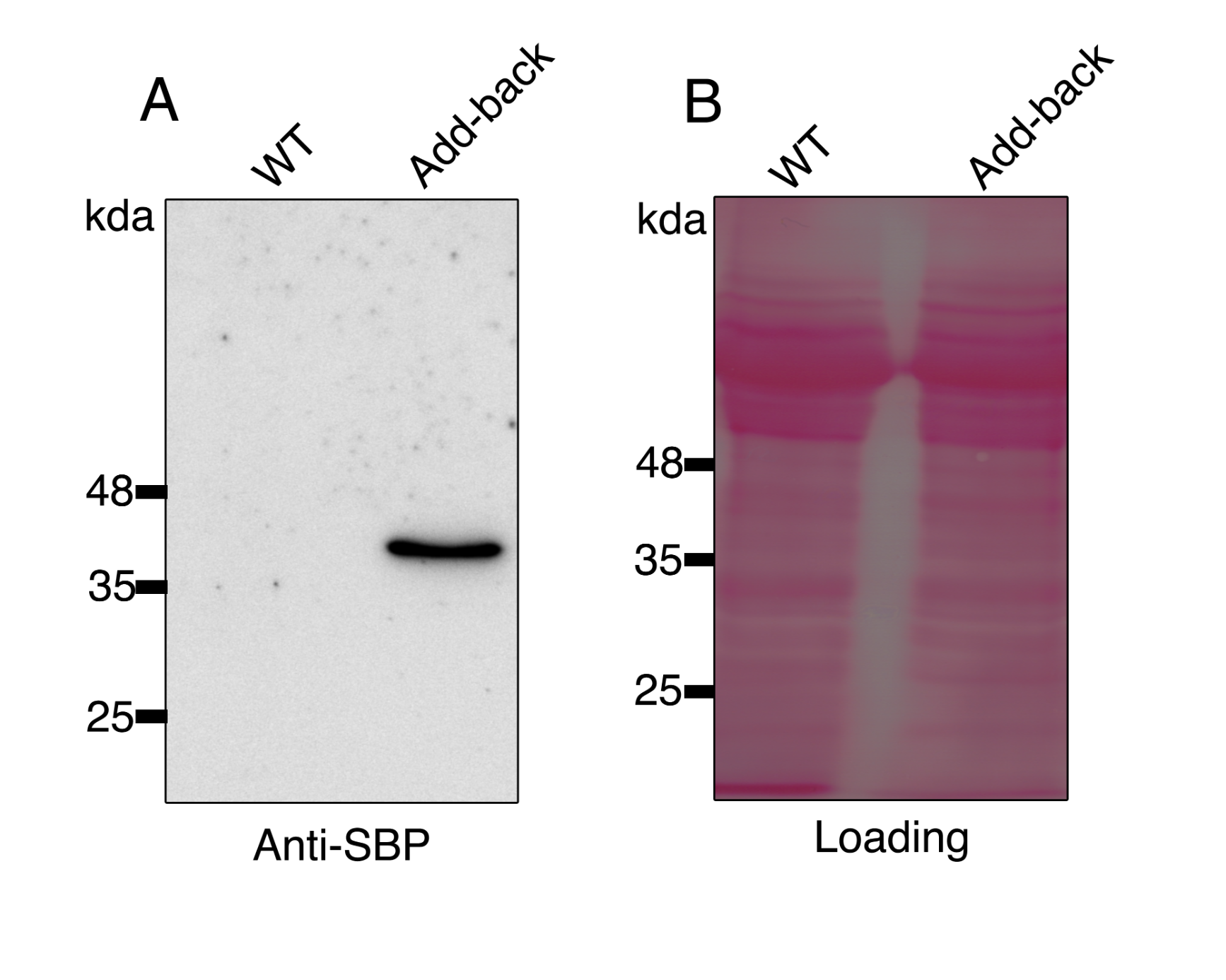

Supplement: S4 Fig — (A) Cell lysates of L. mexicana LeishIF4E2 add-back and WT cells were resolved over 10% SDS-PAGE followed by western analysis with antibodies directed against the SBP tag. (B) Ponceau staining of the blot served as a loading control. (TIF) [file pntd.0008352.s004.tif]

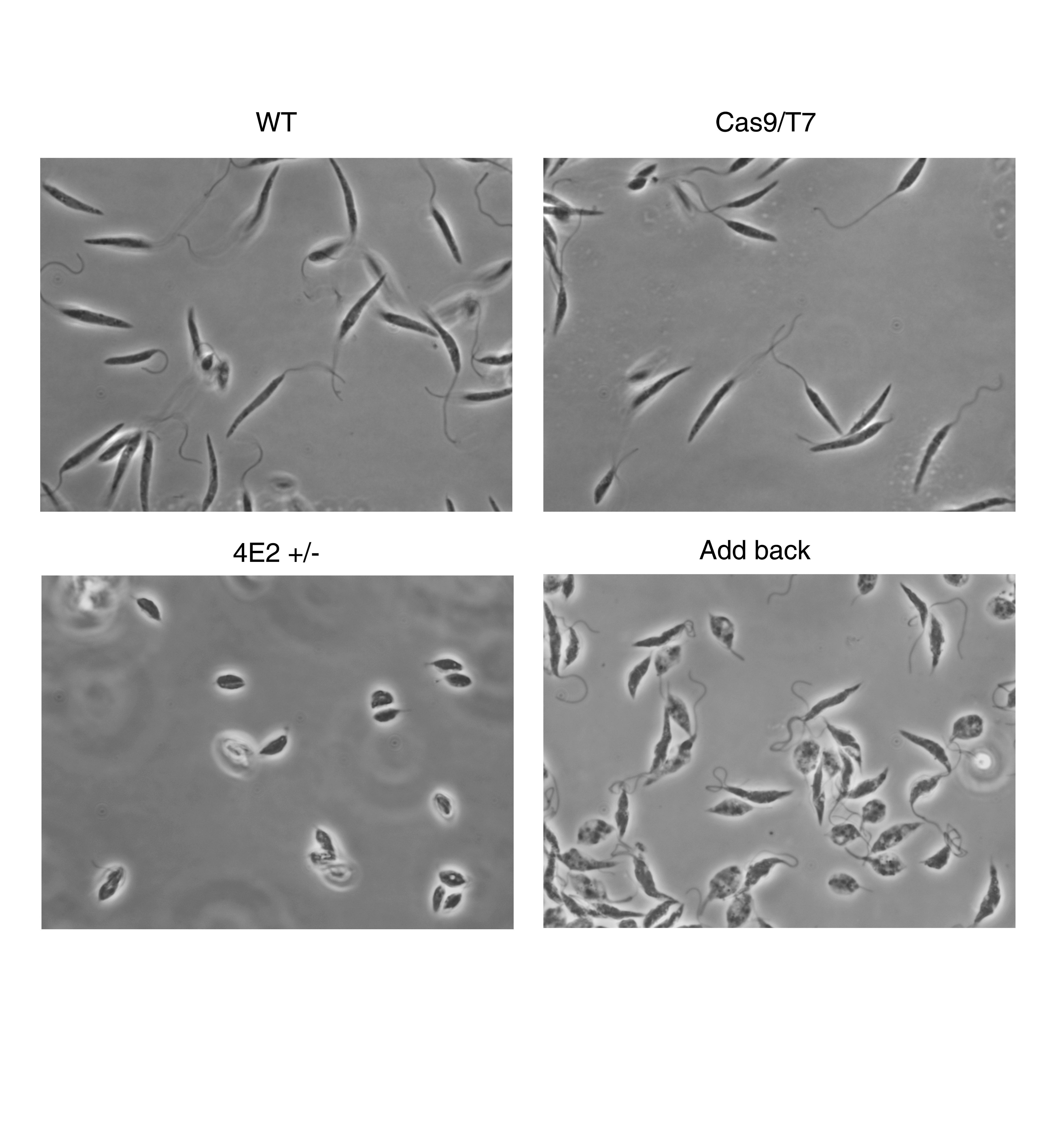

Supplement: S5 Fig — The mutant LeishIF4E2(+/-) mutant, the add-back cells along with WT and Cas9/T7 expresser cells were grown under normal conditions. The cells were fixed, and images were captured at X100 magnification with a Zeiss Axiovert 200M microscope equipped with AxioCam HRm CCD camera. (TIF) [file pntd.0008352.s005.tif]

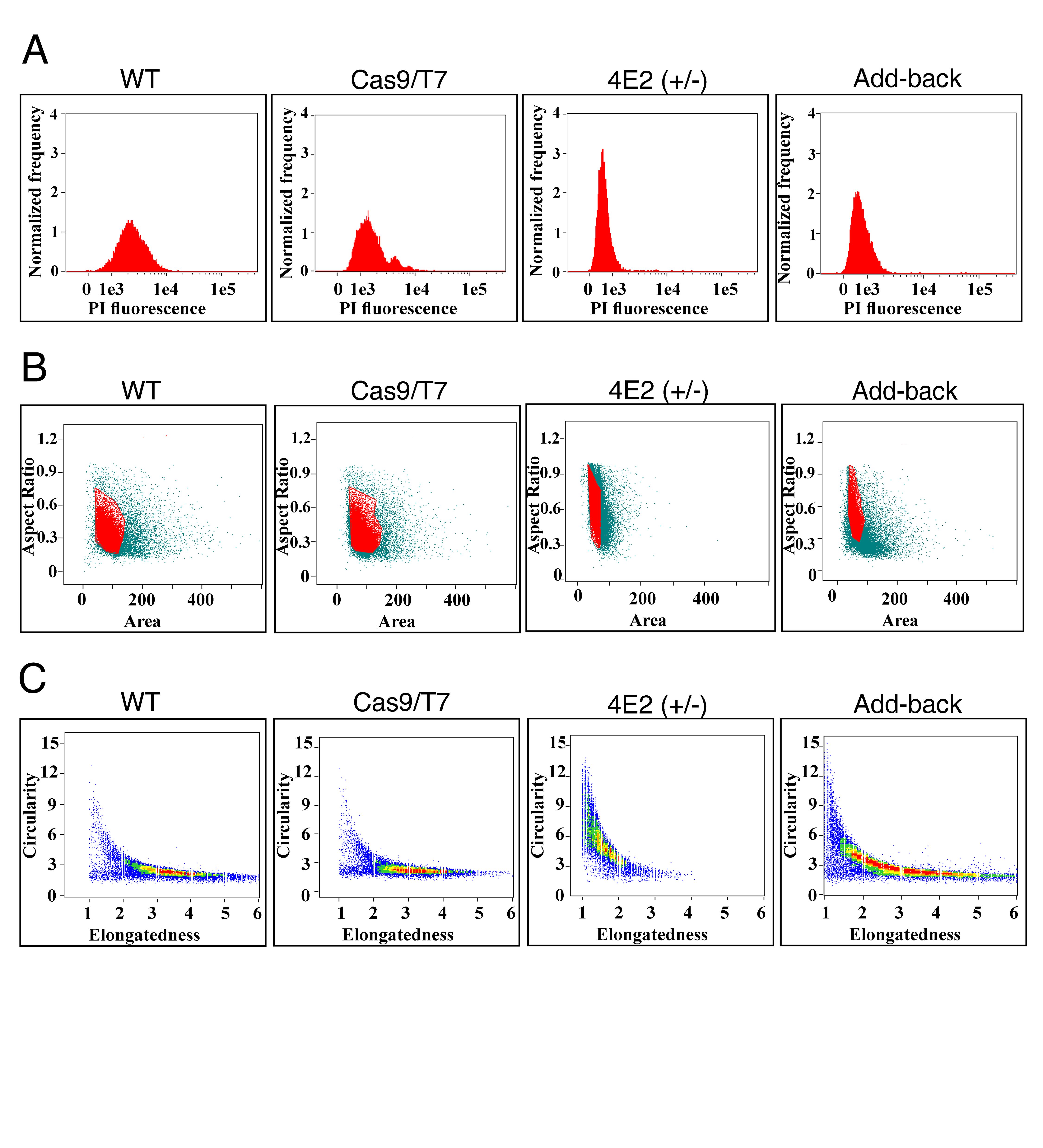

Supplement: S6 Fig — L. mexicana WT, Cas9/T7 expressing control cells, LeishIF4E2(+/-) mutant and add-back promastigotes were subjected to Flow cytometry analysis. (A) Cell viability is represented for focused, single gated cells for all the different cell lines (B) Scatter plots representing gated focused single cell populations for different cell lines. (C) Cell shapes are being represented in terms of circularity or elongatedness as scatter plots for gated cell population. (TIF) [file pntd.0008352.s006.tif]

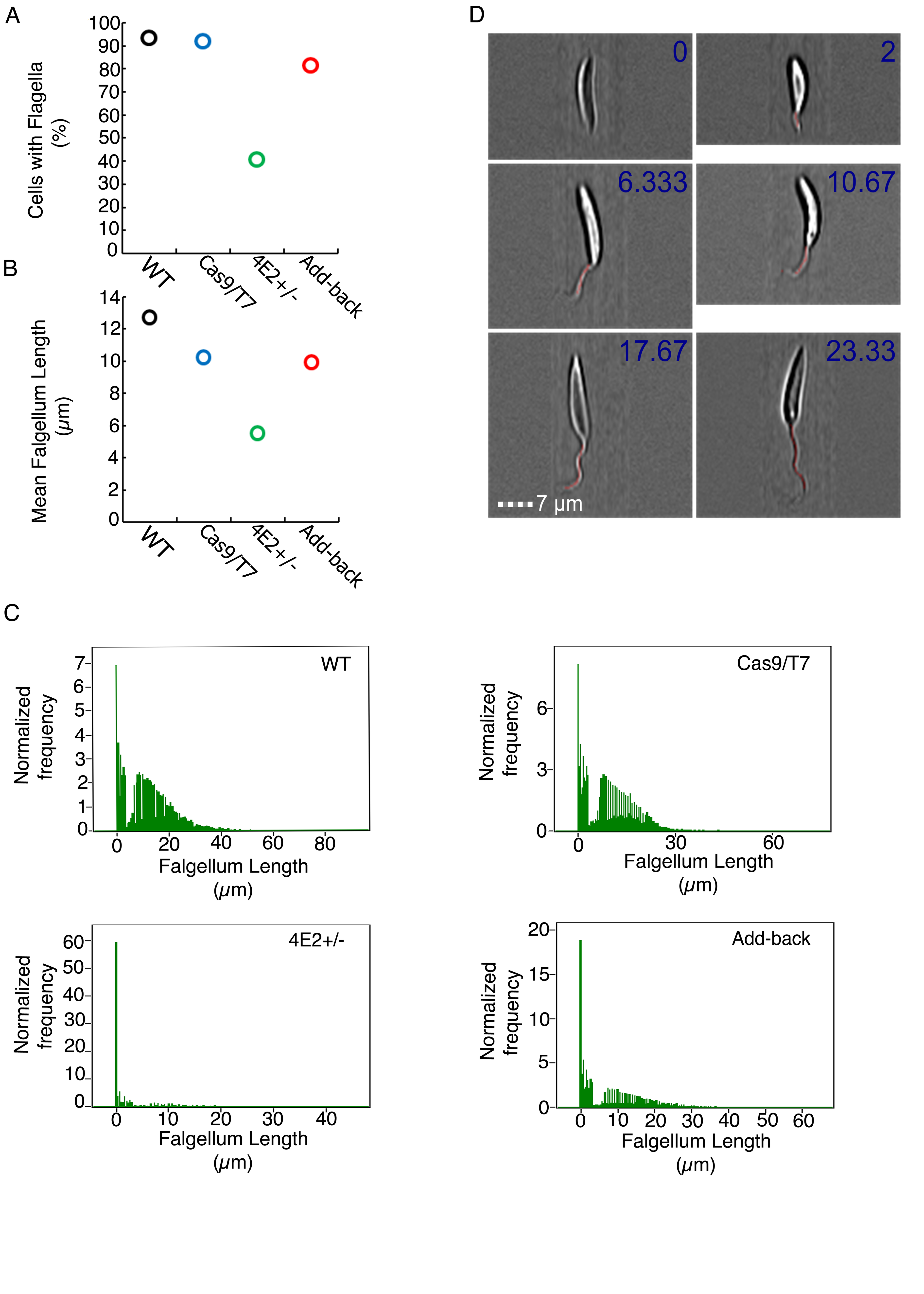

Supplement: S7 Fig — Data were acquired for WT, Cas9/T7, 4E2(+/-) and add-back cells in the assay using ImageStreamX mkII, Objective 60X/0.9NA. (A) An assay containing ~15,000 cells shows the percentage of cells with identifiable flagella (>0 um) as a dot plot. (B) The mean flagellum length of ~15,000 cells is shown as a dot plot. (C) Shows the normalized frequency of flagellar length (D) Representative Brightfield images of cells with various flagella length. Red lines show the mask used to identify the flagellum, numbers in dark blue show flagella length in micrometer for individual images. (TIF) [file pntd.0008352.s007.tif]

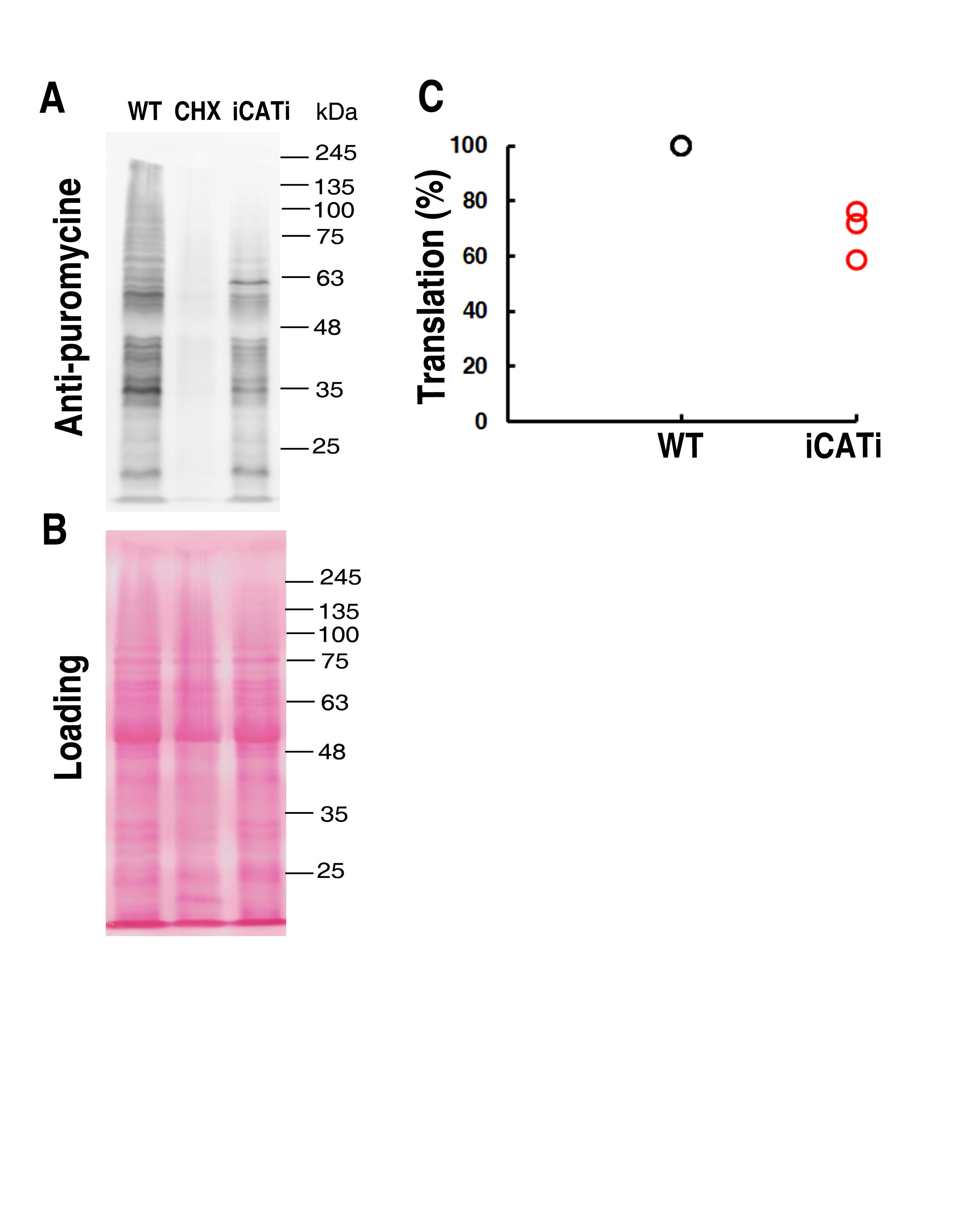

Supplement: S8 Fig — (A) WT and transgenic cells expressing the CAT reporter (iCATi, I represents the HSP83 intergenic region that provides RNA processing signals) were incubated with 1 μg/mL puromycin for 1 hr. Cycloheximide treated cells were used as a negative control for complete inhibition of translation. Puromycin treated cells were lysed and resolved over 12% SDS-PAGE and subjected to western analysis using antibodies against puromycin. (B) Ponceau staining was used to indicate comparable protein loads. (C) Densitometry analysis of puromycin incorporation in the iCATi expressing cell line was compared to wild type (WT) cells (100%). Data from all three independent experiments are represented. (TIF) [file pntd.0008352.s008.tif]

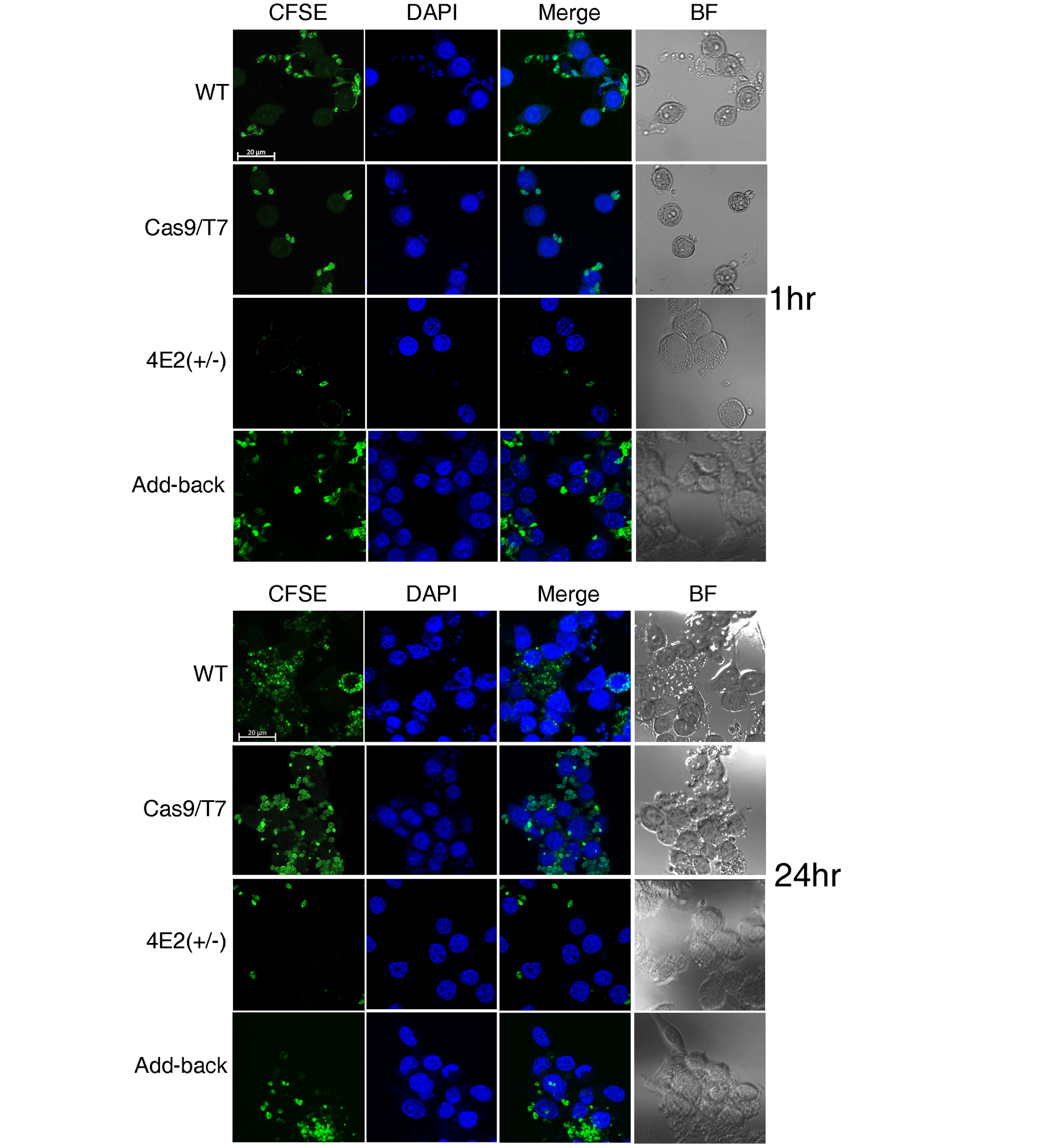

Supplement: S9 Fig — Stationary phase L. mexicana LeishIF4E2(+/-) mutant, WT, Cas9/T7 expressers and add-back cells, were pre-stained with the CFSE dye and used to infect RAW 264.7 macrophages at a ratio of 10:1 for one hour. The cells were then washed to remove excess parasites, and the macrophages were cultured for 1 hr (A) or 24 hr (B) post infection at 37°C. Macrophage nuclei were stained with DAPI and the infected macrophages were processed for confocal microscopy. A representative section of Z-projections (maximum intensity) produced by Image J software is shown. Fields of 200 cells were further evaluated to quantify the infection. (TIF) [file pntd.0008352.s009.tif]

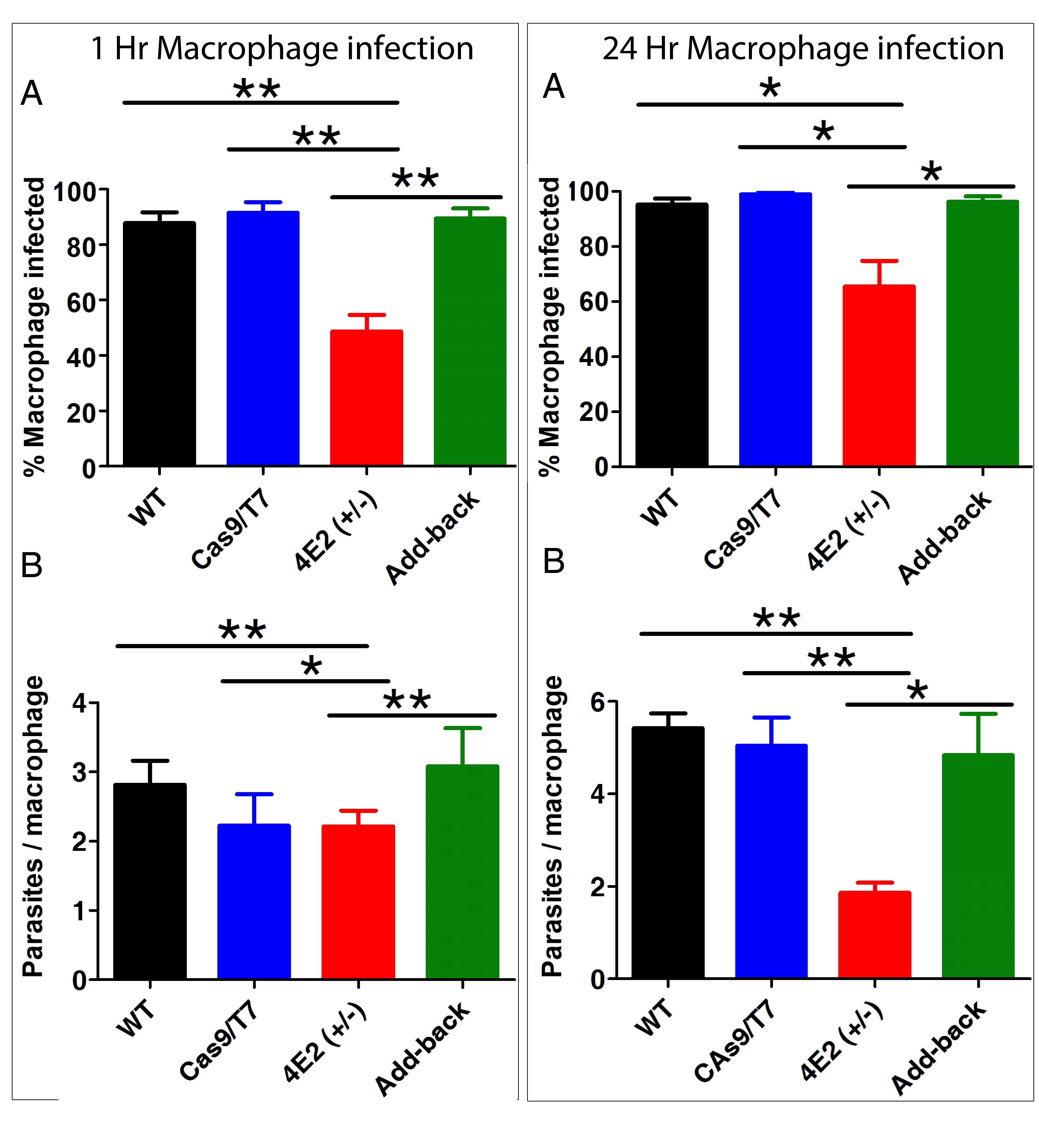

Supplement: S10 Fig — Parasite infectivity of cultured RAW 264.7 macrophages was estimated in vitro using Image J software. (A) The percentage of infected macrophages was determined by counting a total of 100 macrophages from three independent experiments. (B) The average number of parasites per infected cell is shown. Kruskal Wallis test in GraphPad Prism was used to determine the percentage of infected cells and for calculation of the average parasites per cell along with standard deviation values (SD). The percentage of infected macrophages (%) and the average number of parasites per macrophage in the LeishIF4E2(+/-) mutant were compared with each of the control lines: WT, Cas9/T7 expressing cells, the LeishIF4E2(+/-) mutant and the LeishIF4E2 add-back cells. P value < 0.001 is represented by ***, P value < 0.01 by ** and P value < 0.05 by *. The data for 1hr and 24 hr macrophage infections are shown in separate panels. (TIF) [file pntd.0008352.s010.tif]
